# Identification and functional analysis of putative effector proteins from *Diaphorina citri* and ‘*Candidatus* Liberibacter asiaticus’

**DOI:** 10.3389/fpls.2025.1656652

**Published:** 2025-11-27

**Authors:** Sarmina Dangol, Márcio Fernandes Alves Leite, Mônica Neli Alves, Marc Galland, Paula J. M. van Kleeff, Gertjan Kramer, Josiane C. Darolt, Laudecir L. Raiol-Junior, Rafael Brandão Garcia, Leandro Peña, Nelson Arno Wulff, Marcelo Pedreira de Miranda, Silvio Aparecido Lopes, Harro Bouwmeester, Robert C. Schuurink

**Affiliations:** 1 Plant Physiology, Green Life Sciences Research Cluster, Swammerdam Institute for Life Sciences, University of Amsterdam, Amsterdam, Netherlands; 2 Plant Hormone Biology, Green Life Sciences Research Cluster, Swammerdam Institute for Life Sciences, University of Amsterdam, Amsterdam, Netherlands; 3 Fundo de Defesa da Citricultura - Fundecitrus, Araraquara, São Paulo, Brazil; 4 IGEPP, Institute of Genetics, Environment and Plant Protection (INRAE, Institut Agro, University of Rennes), Le Rheu, France; 5 Laboratory for Mass Spectrometry of Biomolecules, Swammerdam Institute for Life Sciences, University of Amsterdam, Amsterdam, Netherlands; 6 Universidade Estadual Paulista (Unesp), Faculdade de Ciências Agrárias e Veterinárias (FCAV), Jaboticabal, São Paulo, Brazil; 7 Instituto de Biologia Molecular y Celular de Plantas – Consejo Superior de Investigaciones Científicas, Universidad Politécnica de Valencia, Valencia, Spain

**Keywords:** citrus greening disease, *Diaphorina citri*, *Candidatus Liberibacter asiaticus*, transcriptomics, proteomics, effectors, reactive oxygen species, plant defense

## Abstract

Phloem feeders, such as the psyllid *Diaphorina citri* (*D. citri*), feed on plants by inserting their stylet bundle followed by probing the apoplast before reaching the phloem. The psyllids secrete watery saliva containing various proteins into the phloem, which can act as effectors to facilitate their feeding or modulate host defense responses. Concomitantly, feeding is the main mode of transmitting the *Candidatus* Liberibacter asiaticus (*C*Las) bacteria to the phloem. *C*Las produces several effectors that have been hypothesized to contribute to Huanglongbing (HLB) virulence. Here, we aimed to identify putative effector proteins from both *D. citri* and *C*Las. To achieve this, we used different omics techniques on different tissues and organs from both plants and insects. More specifically, we performed transcriptomics on the heads of healthy and *C*Las-infected *D. citri* and proteomics of artificial diet and of phloem of four different plant species fed on by healthy and *C*Las-infected *D. citri.* Subsequently, we used various criteria and bioinformatics tools to predict putative effectors. This resulted in the identification of four proteins from *D. citri* [ferritin, prisilkin, CG31997-PA, and pterin-4-alpha-carbinolamine dehydratase-like protein (PCBD)] and two Sec-dependent effectors from *C*Las, CLIBASIA_04560 and CLIBASIA_05320, that were used for further functional studies. The expression of these six proteins in *Nicotiana benthamiana* modified the ROS burst triggered by flagellin, indicating that they can indeed function as effector proteins *in planta*. In addition, expression of the psyllid effectors *in planta* significantly reduced the growth of *Pseudomonas syringae* pv. *tabaci* (*Pta*).

## Introduction

The Asian citrus psyllid (*Diaphorina citri* Kuwayama, Hemiptera: Liviidae) is a phloem-feeding pest of citrus. It is the principal vector of the *Candidatus* Liberibacter asiaticus (*C*Las) bacterium, which is the causal agent of Huanglongbing (HLB), also called citrus greening disease (Grafton-Cardwell et al., 2013). HLB is considered the most serious threat to the global citrus industry (Bové, 2006) due to the challenges with management strategies. Although some genotypes have been reported to exhibit a certain degree of tolerance or resistance to *C*Las (Ramadugu et al., 2016; Alves et al., 2021), no curative treatments or commercially available citrus varieties with confirmed resistance to HLB currently exist. A comprehensive strategy for HLB control must address the pathogen, the vector, and the host. Potential strategies to improve HLB resistance are the evaluation of disease-resistant citrus resources and the identification of virulence factors of both pathogen and its vector and their *in planta* targets for the identification of resistance and susceptibility genes that can be used for breeding purposes (Yang et al., 2024).

Within insect vectors, plant pathogens translocate from the alimentary canal into the hemolymph or other tissues (Hall et al., 2013). The pathogens then move into the salivary glands, contaminate the saliva, and are injected into the host plant during feeding (Weintraub and Beanland, 2006; Hogenhout et al., 2008; Hall et al., 2013). The saliva of Hemiptera is crucial in injecting pathogens into healthy plants during feeding (Weintraub and Beanland, 2006). Interestingly, the proportion of *C*las-infected (*C*Las-positive) salivary glands of *D. citri* was significantly lower than that of other tissues. However, due to their direct role in delivering the pathogen during feeding, the salivary glands are the primary site for *C*Las transmission. This suggests that *C*Las must overcome specific barriers or regulatory mechanisms to reach and colonize the salivary glands, reinforcing the need to identify the mechanisms of salivary colonization and transmission (Hogenhout et al., 2008; Ammar et al., 2011).

In phloem-feeding insects, the saliva plays a critical role in mediating the “warfare” between the insects and their host plants (Cherqui and Tjallingii, 2000). Like other phloem feeders, *D. citri* inserts its stylet bundle and probes intercellularly through the epidermis and parenchyma toward the phloem. During this pathway, the insect secretes a salivary sheath that guides the stylet to the vascular tissue. Unlike aphids and whiteflies, *D. citri* does not puncture or ingest cellular contents before reaching the phloem. After reaching the phloem, *D. citri* performs salivation followed by sap ingestion similar to the behavior of other phloem feeders (Bonani et al., 2010). Saliva contains many bioactive components that function in food digestion, lubrication, tissue penetration, and overcoming plant defenses (Hogenhout and Bos, 2011; Huang et al., 2016). During probing, insects secrete watery saliva containing herbivore-associated molecular patterns (HAMPs) and pathogen-associated molecular patterns (PAMPs) derived from bacteria present in their body or salivary glands (Chaudhary et al., 2014; Jaouannet et al., 2014). When reaching the plant mesophyll cells, upon PAMP or HAMP recognition, plants can trigger local immune responses known as PAMP-triggered immunity (PTI). It can activate complex signaling networks, involving mitogen-activated protein kinase (MAPK) cascades as well as signaling via the jasmonic acid, salicylic acid, or ethylene pathways (Felton and Tumlinson, 2008; Dodds and Rathjen, 2010; Acevedo et al., 2015; Schmelz, 2015). This signaling can lead to a reconfiguration of the transcriptome and proteome, as well as the biosynthesis of defensive chemicals (Wu and Baldwin, 2010; Hogenhout and Bos, 2011).

HAMP-mediated plant defenses may be suppressed by other molecules secreted by herbivores, which are known as effectors (Felton and Tumlinson, 2008; Hogenhout and Bos, 2011). Effectors aid infection by suppressing plant defense, leading to effector-triggered susceptibility (ETS), and create favorable environments for colonization and proliferation (Dodds and Rathjen, 2010; Toruño et al., 2016). Previous studies have implicated a number of salivary-secreted proteins from herbivorous phloem feeders that can act as effectors to facilitate their feeding or repress host immune responses. Examples of this are the proteins C002 and macrophage migration inhibitory factor (MIF) from aphids (Mutti et al., 2008; Naessens et al., 2015); NlEG1 from brown plant hopper (Ji et al., 2017); laccase 1 (LAC1), 2G4, G5 and 6A10, Bt56, and BtFer1 from *B. tabaci* MED (Yang et al., 2017; Lee et al., 2018; Su et al., 2019; Xu et al., 2019); and BSP9, S1, B1, and P1 from *B. tabaci* MEAM1 (Wang et al., 2019; van Kleeff et al., 2024). In general, plants carry receptors, or R (Resistance) proteins, that can recognize effectors, leading to effector-triggered immunity (ETI) (Jones and Dangl, 2006). In plant–insect interactions, recognition of effectors by R-genes is largely unknown. However, gene-for-gene type interactions have been found in several aphid–host systems (Jaouannet et al., 2014). PTI and ETI may be spatiotemporally distinct but are intimately related to the reactive oxygen species (ROS) burst (Grant and Loake, 2000; Torres et al., 2006). Production of ROS in plant cells is a hallmark of successful recognition of plant pathogens and activation of plant defenses (Lamb and Dixon, 1997; Torres, 2010). In plant cells, ROS also occur in response to many physiological stimuli (Mori and Schroeder, 2004; Baxter et al., 2014).

*C*Las is a phloem-limited, Gram-negative bacterium vectored by *D. citri*, which passively inoculates *C*Las directly into the phloem of host plants. *C*Las is the most widespread HLB-causing bacterium, and all commercial citrus varieties are susceptible to it. However, the Oceanian citrus relatives’ genera *Eremocitrus* and *Microcitrus* have been described as resistant or at least less susceptible to the HLB-causing bacterium (*C*Las) (Ramadugu et al., 2016; Alves et al., 2021, 2022). Moreover, even more phylogenetically distant genera, such as *Murraya* and *Bergera*, have been described as a transient host and immune, respectively, to *C*Las (Alves et al., 2021, 2025).

When present in plant mesophyll cells, Gram-negative bacteria mostly secrete effectors directly into host cells through the type III secretion system (Feng and Zhou, 2012). In contrast, *C*Las resides in the sieve elements, which are nuclei-free and have an undefined capacity to generate an effective immune response (Duan et al., 2009; Huang et al., 2020). Moreover, insect- transmitted bacteria, like *C*Las, lack this specialized delivery machinery, but they harbor essential components of the general secretion pathway (SEC translocon) for the delivery of effectors and utilize this system (Sugio et al., 2011). These Sec-delivered effectors (SDEs) carry an N-terminal secretion signal, allowing their export from pathogen cells into the extracellular space.

To date, only a handful of *D. citri* and *C*Las effector proteins have been characterized. So far, using a bioinformatics approach and functional analysis, DCEF32, DcE1, DcitSGP1, or DcitSGP3 has been reported (Pacheco et al., 2020; Kuang et al., 2024; Liu et al., 2024). The silencing of DCEF32 impaired the feeding behavior of *D. citri* on citrus plants (Pacheco et al., 2020). DcE1 modulated plant immunity and enhanced the feeding performance of *D. citri* (Kuang et al., 2024). DcitSGP1 and DcitSGP3 enhanced *D. citri* feeding by suppressing jasmonic acid-mediated defense responses in citrus (Liu et al., 2024). In addition to this, some proteomic and transcriptomic analyses of saliva and salivary glands of *D. citri* have been reported (Liu et al., 2020; Wu et al., 2021). In *C*Las, 27 core SDEs that are conserved across various strains have been identified (Thapa et al., 2020). Several SDEs interact with different host targets and putatively interfere with citrus immune responses. For example, SDE1 interacts with the papain-like cysteine proteases (PLCPs), attenuates PLCP activity, and contributes to HLB progression (Clark et al., 2018, 2020). SDE15 inhibits plant immunity and promotes the colonization of *C*Las by interacting with citrus ACD2 (ACCELERATED CELL DEATH 2) (Pang et al., 2020). Still, many SDEs remain to be characterized.

Host–pathogen relationships involve a myriad of molecular interactions that play important roles both in a pathogen’s ability to suppress or avoid a plant’s immunity and the host’s ability to detect and resist the pathogen. Thus, studying this interplay between host, vector, and pathogen is vital for understanding and eventually enabling the control of HLB disease. *Diaphorina citri* and *C*Las are the main contributing agents of HLB disease, but little is known about their effector biology. The aim of our study is to identify effector proteins from both *D. citri* and *C*Las using transcriptomics and proteomics approaches. To achieve this, we used heads of *D. citri* with and without *C*Las for transcriptome analyses. In addition, artificial diet fed on by psyllids, infected with or free of CLas, and phloem exudates of different host plants infected by *C*Las or infested by *D. citri* were used for proteomics.

## Materials and methods

### Collection of insects

The insect colonies were maintained at Fundecitrus (Fundo de Defesa da Citricultura), Araraquara - SP, Brazil. A *C*Las-free *D. citri* colony was maintained in cages on healthy *Murraya paniculata* L. (Jack) plants. Healthy and *C*Las-infected sweet orange [*Citrus × sinensis* (L.) Osbeck grafted on ‘Rangpur’ lime (*C. × limonia* Osbeck)] were maintained in 4-L bags in a greenhouse and grown in decomposed pine bark substrate (Plantmax citrus, Eucatex, Paulínia, SP, Brazil). Both healthy and *C*Las-infected plants were pruned and maintained under controlled conditions: temperature of 26°C ± 2°C, relative humidity of 70%, and photoperiod of 14/10 h of light/dark for shoot development and experimentation. For *C*Las acquisition, *C*Las-free *D. citri* were confined and kept on citrus shoots at the vegetative stage V2/V3 of *C*Las-infected sweet orange plants for 7 to 10 days (Cifuentes-Arenas et al., 2018; Lopes and Cifuentes-Arenas, 2021). Similarly, to obtain *C*Las-free *D. citri* (healthy), *C*Las-free *D. citri* from *M. paniculata* were confined and kept on citrus shoots at the vegetative stage V2/V3 of *C*Las-source plants for 7 to 10 days. After confinement, the presence of *C*Las on *D. citri* was confirmed by performing quantitative polymerase chain reaction (qPCR) on four insects randomly sampled from each *C*Las-source plant (Li et al., 2006).

### RNA extraction

The dissection of heads of psyllids with salivary glands was carried out after 7 days of acquisition-access on *C*Las-source plants or healthy ‘Valencia’ plants. In total, 200 heads with salivary glands were separated from *C*Las-infected and healthy *D. citri*. Five repetitions were performed for each acquisition period on *C*Las-source plants and healthy ones. The dissected head samples were transferred into 500 µL of RNA Later (Invitrogen/Thermo Fisher Scientific, Waltham, USA) on ice. Total RNA extraction was performed using RNeasy Lipid Tissue Kit (Qiagen, Venlo, the Netherlands) according to the manufacturer’s protocol. Samples were resuspended in 30 µL of nuclease-free water. Total concentration was measured using a NanoDrop (Thermo Fisher Scientific, Waltham, MA, USA).

### RNA-seq and bioinformatic analysis

RNA integrity was examined using the 2200 TapeStation System with Agilent RNA ScreenTapes (Agilent, CA, USA). A total of 10 RNA samples (five *C*Las-infected and five *C*Las-free *D. citri*) with RIN values greater than 5 were used for paired-end Illumina HiSeq 2000 sequencing (HiSeq 2000). Reads were cleaned from adapters and ambiguous sequences by Trimmomatic 0.32 software (Bolger et al., 2014).

The complete RNA-seq analysis was performed using version 0.3.7 of a custom Snakemake pipeline (https://f1000research.com/articles/10-33/v2) that combines fastp v0.19.5 for read trimming (PMID: 30423086), STAR v2.7.6a splice-aware aligner (PMID: 23104886), subread v1.6.0 for quantification (PMID: 23558742), and multiQC v1.9 for quality checks (PMID: 27312411). Differentially expressed genes (adjusted *p*-value < 0.05, Benjamini and Hochberg method) were called using DESeq2 on the raw counts and the presence/absence of *C*Las as an explanatory factor in the DESeq2 model.

The Snakemake pipeline code is available on Zenodo (zenodo.org/records/4707140) and GitHub (bleekerlab/snakemake-rnaseq). RNA-seq reads are deposited at the European Nucleotide Archive under project number PRJEB46772. Complete analysis is available on GitHub (https://github.com/SchuurinkLab/diaphorina_citri_effectors/releases/tag/v0.1.0).

### Cloning and *Agrobacterium* transformation of putative effectors

The coding region of effector proteins, with and without signal peptide, was amplified from cDNA of *C*Las-infected psyllids prepared using the SuperScript III kit (Invitrogen, Waltham, USA) using gene-specific primers containing Gateway attB1 and attB2 sites (**Supplementary Table 1**). The primers were generated using sequence information from citrusgreening.org. The amplified coding regions were subcloned into pDONR221 entry vector using BP clonase (Invitrogen, Waltham, USA) to create entry clones. The entry clones without signal peptide were recombined into Gateway destination vectors pGWB452, 35S_pro_: N-terminal fusion with GFP and without tag (pGWB502, 35S_pro_): (Nakagawa et al., 2017) using LR clonase (Invitrogen, Waltham, USA) for subcellular localization and ROS assay, respectively. Similarly, the entry clones with signal peptides were recombined into the Gateway destination vector pGWB502 using LR clonase (Invitrogen) for *Pseudomonas syringae* pv. tabaci (*Pta*) assays. Competent DH5 alpha *Escherichia coli* cells were transformed with the constructs and selected on Luria–Bertani (LB) agar plates containing 50 μg/mL of spectinomycin. Colony PCR and subsequent Sanger sequencing were used to confirm the correct sequence of the effectors.

Electroporation was used to transform 50 μL aliquots of competent *Agrobacterium tumefaciens* (strain GV3101) cells with 1 μL of each plasmid (100–200 ng of DNA) containing a gene of interest. Following recovery in non-selective LB-low salt liquid medium (NaCl 5 g/L) for 2h at 28°C, cells were plated on selective LB-agar containing 50 μg/mL of spectinomycin, 25 μg/mL of rifampicin, and 50 μg/mL of gentamicin and grown for 48h at 28°C. The correct colonies were selected by colony PCR using gene-specific primers.

### Plant material and transient expression

*Nicotiana benthamiana* plants were grown under greenhouse conditions (21°C, 16/8h photoperiod) for 5 to 6 weeks. One day prior to infiltration, the plants were transferred to a greenhouse compartment with natural daylight (20°C). A single colony of the controls [cyan fluorescent protein (CFP); green fluorescent protein (GFP); NLS:mCherry] or candidate effectors and a silencing suppressor (P19) from *A. tumefaciens* GV3101 were used to inoculate 10 mL aliquots of selective LB- low salt liquid medium containing selective antibiotics in universal tubes and grown overnight at 28°C at 200 rpm. The cells were collected by centrifugation at 3,000 rpm for 10min, resuspended in 10 mL of infiltration buffer [5 g/L of Murashige & Skoog Basal Salt Mixture without vitamins (Duchefa, the Netherlands), 20 g/L of sucrose, 10 mM of 2-(*N*-morpholino) ethanesulfonic acid (MES) (pH 5.6), and 200 μM of acetosyringone]. For subcellular localization, we used an OD_600_ of 0.2, and for ROS assays, we used an OD_600_ of 0.4. Cultures of candidate effectors or controls were mixed in a 1:1 ratio with the silencing suppressor P19 and infiltrated into the abaxial site of the third or fourth leaf of *N. benthamiana* plants using a needleless syringe.

### Reactive oxygen species production assay induced by flg22

ROS production was determined using a luminol-based assay as described in Keppler et al. (1989). Briefly, *A. tumefaciens* GV3101 containing candidate effectors without the signal peptide and the controls CFP or GFP were infiltrated into *N. benthamiana*. One-half of the leaf was infiltrated with CFP/GFP with the silencing suppressor P19 (control), and the other half of the leaf was infiltrated with a candidate effector with the silencing suppressor P19. The plants were placed for 48h in a greenhouse with natural daylight at 20°C for expression. Leaf discs of 16 mm^2^ were collected with a cork borer from the *N. benthamiana* infiltrated spots and transferred to 96-well white plates. Assays were performed on eight plants, and from each infiltration spot, three replicates were taken. The leaf discs were floated for 12h on 190 μL autoclaved MQ water to eliminate wound-induced ROS. After incubation, the water was replaced by a mixture of 100 nM of flg22 (QRLSSGLRINSAKDDAAGLAIS; Felix et al., 1999), 0.–5 mM of luminol probe 8-amino-5-chloro-7-phenyl-pyrido[3,4-d]pyridazine-1,4(2H,3H)dione (L-012) (Wako Chemicals, Richmond, VA, USA), and 20 μg/mL of horseradish peroxidase type VI-A (Sigma, Saint Louis, MO, USA). ROS production was measured by using the Synergy HTX Multimode Reader (Agilent, CA, USA) over 45 =min with measurement intervals of 2 =min, with an integration time of 1 s. Eight biological replicates were used. Statistical analyses of the data obtained were performed using Prism 7 software (GraphPad, Boston, Massachusetts, USA). The data are represented as means ± SD. Statistical significance was analyzed by the Student’s *t*-test.

### Subcellular localization

*Agrobacterium* (strain GV3101) carrying GFP-tagged candidate effector constructs or mCherry tagged with a nuclear localizing signal (NLS-mCherry) was grown for 1 day at 28°C in LB medium supplemented with 25 μg/mL of rifampicin, 50 μg/mL of gentamicin, and 50 μg/mL of spectinomycin for GFP-tagged constructs and with 25 μg/mL of rifampicin, 50 μg/mL of gentamicin, and 50 μg/mL of kanamycin for the NLS-mCherry construct. The agrobacteria carrying the gene of interest fused to a fluorescence tag were infiltrated together with the P19 silencing inhibitor construct in infiltration buffer with an OD_600_ of 0.2, on the abaxial leaf side of 4–6-week-old *N. benthamiana* plants. *N icotiana benthamiana* leaves were infiltrated as described above. Expression was allowed for 2 days at 21°C under a 16-h/8-h light–dark regime. Just prior to imaging, discs were taken from the infiltrated spots and placed on glass slides using a 16-mm^2^ cork borer. Single-plane imaging of the fluorescence signal was performed on a Leica SP8 confocal microscope using a ×20 water objective lens. mCherry was excited with a wavelength of 561 nm, and emission was detected at 592–632 nm. GFP was excited with a wavelength of 440 nm, and emission was detected at 465–500 nm. Autofluorescence of chlorophyll was detected at 657–737 nm.

### Artificial diet collection

The artificial diet was collected as described by Hall et al. (2010). Briefly, the base diet was prepared using 15% sucrose plus 0.1% green and 0.4% yellow food color in tap water. Then, in five small plastic tubes (3cm in height and 2cm in diameter), 15 *C*Las-infected and 15 non-infected *D. citri* were transferred. Around a 2.5 × 2. 5-cm piece of Parafilm membrane was stretched until it became a thin layer and placed on top of the vial with *D. citri*, followed by pipetting 250 µL of diet at the center of the membrane. A second membrane was then stretched across the first, sandwiching the diet. All *D. citri* were fed on an artificial diet for 48h under the conditions of 25 °C ± 2 °C, 60% ± 10% R.H., and 14L:10D h photoperiod. The diets fed on by *C*Las-infected and *C*Las-free psyllids were then collected in 2 mL microcentrifuge tubes. The experimental setup was repeated four times. The collected artificial diet was concentrated using the acetone precipitation method. Briefly, four volumes of acetone were added to the artificial diet and incubated at −20°C for an hour. The pellet was separated by centrifugation at 15,000 × *g* for 10min and then dried. After evaporating acetone, the pellet was dissolved in 10 µL of Ambic buffer (100 mM of ammonium bicarbonate, 10 mM of tris(2-carboxyethyl) phosphine, 40 mM of chloroacetamide).

### Phloem extraction

The *D. citri*- and *C*Las-susceptible hosts *Citrus × sinensis* ‘Valencia’ (sweet orange) and *Murraya paniculata* (orange jasmine, a *C*Las-transient host), along with the immune *Bergera koenigii* (curry leaves) and the *C*Las-resistant *Eremocitrus glauca* (Australian desert lime), were drastically pruned and maintained under controlled conditions as described above. While ‘Valencia’ sweet orange, orange jasmine, and curry leaves are all susceptible to psyllid, Australian desert lime is reported to have low nymph viability. ‘Valencia’ sweet orange and Australian desert lime plants were propagated onto ‘Rangpur’ lime rootstock, while the non-compatible orange jasmine and curry leaf plants were grown from seeds. Four 3-year-old plants from each selected genotype were pruned, and adult *C*Las-infected insects (15–20 days old) were confined to the newly developed V2/V3 flushes. The *C*Las-infected psyllids were then maintained on the flushes of all plants at the vegetative stage V2/V3 for 2, 6, and 30 days. After 2 and 6 days of feeding, insects were removed, and phloem was extracted from the flushes. At 30 days, the phloem was extracted from the bark, following the methods described by Killiny (2019), with slight modifications. Simultaneously, four additional plants from each genotype, maintained under the same conditions, were exposed to *C*Las-free psyllids and kept without contact with insects. All plant sampling followed the same procedure.

After 2 and 6 days of being exposed to *C*Las-infected, *C*Las-healthy psyllids, or no psyllids, flushes were excised from all plants, and the petioles were immersed in 1 mL of bleeding buffer (5 mM of phosphate buffer, 5 mM of EDTA) overnight in humid conditions (26°C ± 2°C and 70% of relative humidity). After 30 days of psyllid confinement, the outer bark from mature flushes was removed and immersed in 1 mL of 5 mM EDTA overnight in humid conditions (26°C ± 2°C and 70% of relative humidity). The presence of sucrose in the phloem exudates was confirmed by Benedict’s test (Benedict, 1909). Proteins in the exudates were then concentrated by acetone precipitation Thermo Fisher Scientific (2009) and suspended in 100 µL of Ambic buffer (100 mM of ammonium bicarbonate, 10 mM of tris(2-carboxyethyl) phosphine, 40 mM of chloroacetamide). All samples were quantified using the Bradford protein assay according to the manufacturer’s protocol (Thermo Fisher Scientific, Waltham, USA).

### Sample preparation for LC-MS analysis

The protein samples from the artificial diet and phloem exudates were reduced and alkylated in one step by incubation with tris-(2-carboxyethyl)phosphine and chloroacetamide (Sigma-Aldrich, USA) at the end concentrations of 10 mM and 30 mM, respectively, for 30 min at 70°C. Subsequently, samples were prepared for mass spectrometry analysis using the single-pot, solid-phase-enhanced sample preparation (SP3) protocol (Hughes et al., 2014), with modifications to optimize soluble protein recovery. This optimization entailed that no detergents were added to the samples to enable optimal precipitation of soluble proteins, and the precipitation time was extended to 30 min at room temperature. Following washes, beads were air-dried and resuspended in 100 mM of ammonium bicarbonate (Sigma-Aldrich, USA), after which trypsin (sequencing grade-modified, Promega, Madison, WI, USA) was added at a protease-to-protein ratio of 1:50 (w/w) at 37°C. Following overnight digestion, formic acid was added to achieve a final concentration of 1%, resulting in an approximate pH of 2. The samples were then placed on a magnetic separator device, and the peptides were recovered for LC-MS analysis.

#### LC-MS analysis for quantitative proteomics

The resulting samples were separated by reverse-phase chromatography using an Ultimate 3000 RSLCnano UHPLC system (Thermo Scientific, Germering, Germany). Peptide separation was performed on a 75-μm × 250-mm analytical column (C18, 1.6 μm particle size, Aurora, Ionopticks, Australia), maintained at a temperature of 50°C and operated at a flow rate of 400 nL/min with 3% solvent B for 3 min (solvent A: 0.1% formic acid in water, solvent B: 0.1% formic acid in acetonitrile, ULC-MS-grade, Biosolve, Valkenswaard, The Netherlands). Following this, a multistage gradient was applied, with 17% solvent B at 21 min, 25% solvent B at 29 min, 34% solvent B at 32 min, and 99% solvent B at 33 min, and kept at 99% solvent B till 40 min. The system was returned to initial conditions at 40.1 min and was held until 58 min for equilibration. The eluted peptides were electrosprayed by a captive spray source via the column-associated emitter and were analyzed by a TIMS-TOF Pro mass spectrometer (Bruker, Bremen, Germany). The instrument was operated in PASEF mode for standard proteomics acquisition. PASEF MS/MS scans were initiated 10 times with a total cycle time of 1.16 s, a target intensity of 2e4, an intensity threshold of 2.5 × 10^3^, and a charge state range of 0–5. Active exclusion was enabled for a period of 0.4 min, with precursors being reevaluated if the ratio of the current intensity to the previous intensity exceeded 4. The data from the artificial medium and phloem exudates are deposited at Proteome Xchange under project number PXD062026.

#### Spectral data processing and proteome database search

LC-MS data were processed using the MaxQuant software (version 1.6.14.0) using standard settings, i.e., trypsin/p as the enzyme allowing for two missed cleavages with carbamidomethylation at cysteine as a fixed modification and oxidation at methionine as a variable modification searching the proteome databases of *Citrus_sinensis*, *Diaphorina citri*, and *Candidatus* Liberibacter Asiaticus (UniProt downloaded June 2021). MaxQuant outputs were used for subsequent analysis using the Perseus (version 1.6.1) software. Biological processes, molecular functions, subcellular locations, and protein pathways were annotated with Gene Ontology and KEGG.

### Proteomics data analysis

The analysis of proteomics data combined classic multivariate approaches with generalized joint models. First, the classic multivariate analysis aimed to estimate the capacity of the different factors of the experimental design (genotype, infection, and days after infection) to explain the variability of the proteomics data. This was done by using the centered log-ratio transformed table of intensity and analyzing it using principal component analysis, followed by between-class analysis (BCA). BCA allows measuring the variance of the entire dataset restricted to one grouping factor (Chessel et al., 2004). BCA analyses were performed to measure the variance related to the individual effects of design (genotype, infection, and days after infection) and their combinations.

In parallel, the proteomics dataset was also analyzed using a model-based approach to seek treatment-specific responses and potential interactions between them. For that, we used the generalized joint attribute modeling (GJAM) from the gjam R package (Clark et al., 2017). GJAM allowed us to model the experimental design as a split-plot in time and evaluate the proteomics data under the compositionality constraints. Mass spectrometry exhibits a fundamental compositional nature due to the limited set of ions analyzed in each spectrum. This restriction serves a crucial purpose, ensuring that ions within the confines of a mass spectrometer consistently and predictably respond to electrical fields. Therefore, to explore the effect of treatments in the proteomics data, we consider its compositional nature in the model. GJAM is based on Bayesian statistics and uses Gibbs sampling to estimate the regression coefficients. We used a burn-in of 10,000 and 50,000 iterations to reach stability of the estimates. For complete details on model specification, see Clark et al. (2017). We focus on the interpretation of protein-specific effect sizes for each parameter and group them according to their response. For every treatment, we compared the changes induced by the *D. citri* -only and the *C*Las-positive *D. citri* treatment, having the healthy plant as the reference level (control treatment). Depending on the first time point in which protein levels showed a significant shift, we grouped them into fast response (significant change already detected at the first time point —1 dpi) and late response (significant change detected after the second or third time point).

### *Pseudomonas syringae* infection

The *Pseudomonas syringae* infection was performed as described previously (Lee et al., 2018). Briefly, the leaves of 5-week-old *N. benthamiana* plants were infiltrated with *Agrobacterium* carrying psyllid effectors with the signal peptide, the positive control, *Bemisia tabaci* effector 2G5 (Lee et al., 2018), or as a negative control, the empty vector. Pta was cultured in King’s B plates with 100 μg/mL of rifampicin at 28°C overnight. The next day, Pta was scraped off the plates and resuspended in 1 mL of 10 mM MgCl_2_ in Eppendorf tubes. After centrifugation at 1,000 rpm for 2min, the bacterial cells were resuspended in 10 mM of MgCl_2_ to a final optical density of 0.01 (OD_600_=0.01). After 2 days, the infiltrated leaves were subsequently infiltrated with Pta. Two 1-cm-diameter leaf discs covering the infiltration sites were excised after 1 h and 3 days of Pta inoculation, ground, and suspended in 1 mL of 10 mM MgCl_2_, and 10 µL of serial dilutions of bacterial solution were spread onto selective medium (King’s B agar medium containing 100 μg/mL of rifampicin) and incubated for 2 days in a 30°C growth chamber. Five biological replicates were used. The experiments were repeated three times. Statistical analyses of the data obtained were performed using Prism 7 software (GraphPad). The data are represented as means ± SD. Statistical significance was analyzed by one-way ANOVA (Tukey’s test).

## Results

### RNA-seq prediction of putative effector proteins from the heads of healthy and *C*Las-infected *Diaphorina citri*

In the Asian citrus psyllid, *D. citri* Kuwayama, *C*Las propagates in salivary glands and the midgut. Differential gene expression analysis of *C*Las-infected psyllids showed that *C*Las alters the gene expression profile of the gut, salivary glands, and whole body (Yu et al., 2020; Liu et al., 2021). Moreover, gene expression analysis of *C*Las-infected and *C*Las-free nymphs and adults showed that expression of some of the known psyllid effector genes is modulated in response to Liberibacteria (Pacheco et al., 2020). As expected, *C*Las likely manipulates the physiology of its vector to enhance its own transmission or alters effector expression to modulate defenses of the host plant to facilitate bacterial colonization. Such changes include both upregulation and downregulation, depending on whether the effector promotes compatibility with the host plant or responds to *C*Las-induced stress within the insect.

**Figure 1:**
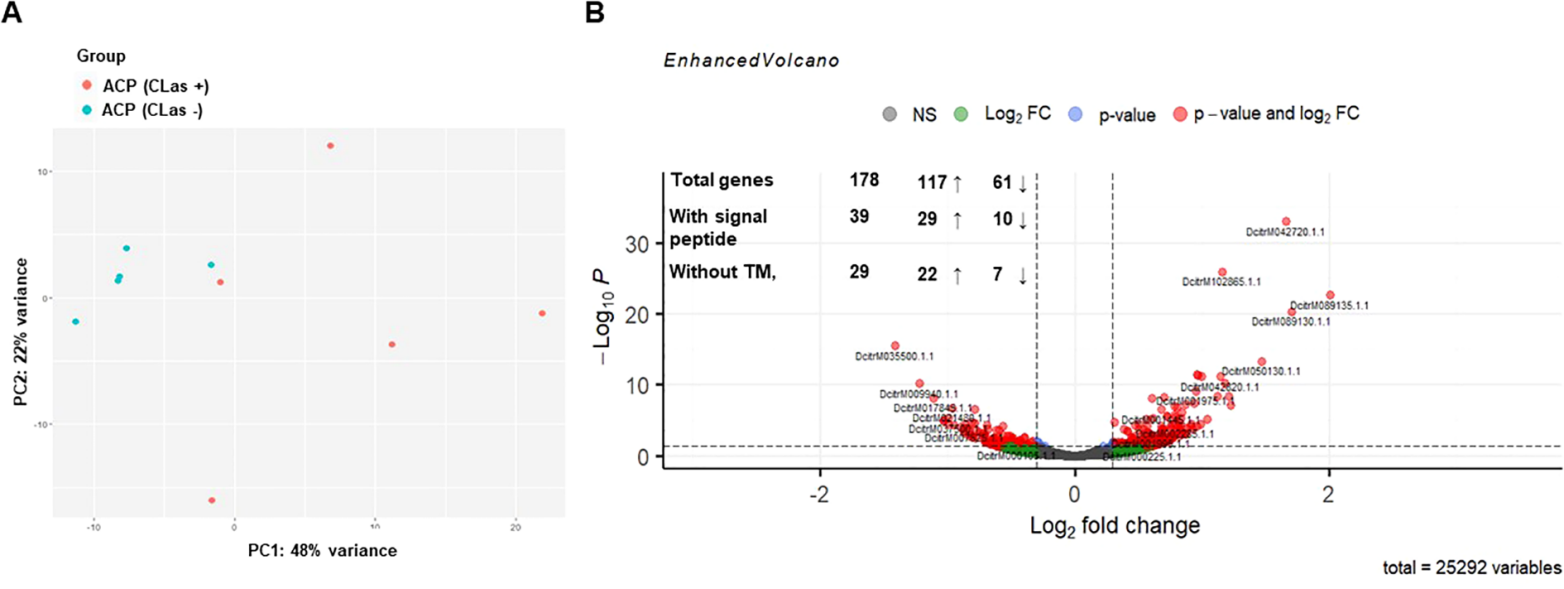
Transcriptional analysis of heads of healthy and *C*Las-infected *Diaphorina citri*. **(A)** Principal components analysis of transcriptome data. Each point represents an RNA-seq sample. Samples with similar gene expression profiles are clustered together. Sample groups are indicated by using different colors as indicated in the legend provided. **(B)** Volcano plot showing the differential expression analysis results for the RNA-seq dataset and the number of differentially expressed genes (DEGs), genes obtained after screening for the presence of the signal peptide (SignalP 4.1) and the absence of the transmembrane domain (TMHMM Server, v. 2.0). The up (↑) and down (↓) arrows with numbers represent up- and downregulated *D*. *citri* genes by *C*Las.

To identify new effectors of *D. citri* related to the presence of *C*Las, RNA-seq analysis was performed on head samples containing salivary glands of healthy and *C*Las-infected *D. citri*. Pairwise comparison between healthy and *C*Las-infected *D. citri* resulted in 178 genes that were differentially expressed in *C*Las-infected ones (*P* ≤ 0.01; **Figure 1**, **Supplementary Table 1**), of which 117 were up regulated (Log_2_FC > 0) and 61 were downregulated (Log_2_FC < 0). Considering that insect effectors are mostly secreted through the classical eukaryotic endoplasmic reticulum (ER)-Golgi pathway through salivary glands, we subsequently selected proteins predicted to have a signal peptide but no transmembrane (TM) domains using SignalP 4.0 and TMHMM v2, respectively. Out of these 178 proteins, 39 had a signal peptide, of which 29 were up regulated and 10 were downregulated. In addition, of these 39 proteins, 29 lacked transmembrane domains, of which 22 were upregulated and 7 were downregulated (**Figure 1**, **Supplementary Table 2**).

From the 29 candidate proteins identified in the *C*Las-infected *D. citri* heads, we applied three main criteria to select candidates for functional analysis: i) strong expression in the RNA-seq dataset, ii) presence of conserved protein domains associated with host manipulation, and iii) specificity to the *D. citri* genome to reduce the risk of off-target effects in the host. Based on these criteria, we selected the following three proteins: DcitrM013325.1.1 (prisilkin-39-like/prisilkin), DcitrM093305.1.1 (CG31997-PA), and DcitrM068575.1.1 (pterin-4-alpha-carbinolamine dehydratase-like protein; PCBD) for functional analyses. These had not only high expression values (**Supplementary Table 2**) but also interesting functional domains as protein binding or transcriptional modulation, or structural flexibility. Prisilkin-39 is predicted to be a glycine-rich protein, and CG31997-PA has a single domain von Willebrand factor type C. PCBD has 4-alpha-hydroxytetrahydrobiopterin dehydratase activity, and it can function both as a transcriptional coactivator and pterin dehydratase (Mistry et al., 2021). Insect effectors are often species- or genus-specific (Boulain et al., 2018). PCBD has 80% amino acid similarity with whitefly (*Bemisia tabaci)* PCBD, but no orthologs were found for prisilkin and CG31997-PA in other phloem feeders, giving a notion that they are specific to *D. citri*. These three putative effectors were used for further functional analysis.

### Identification of putative effector proteins from the saliva proteome of healthy and *C*Las-infected *Diaphorina citri*

**Figure 2:**
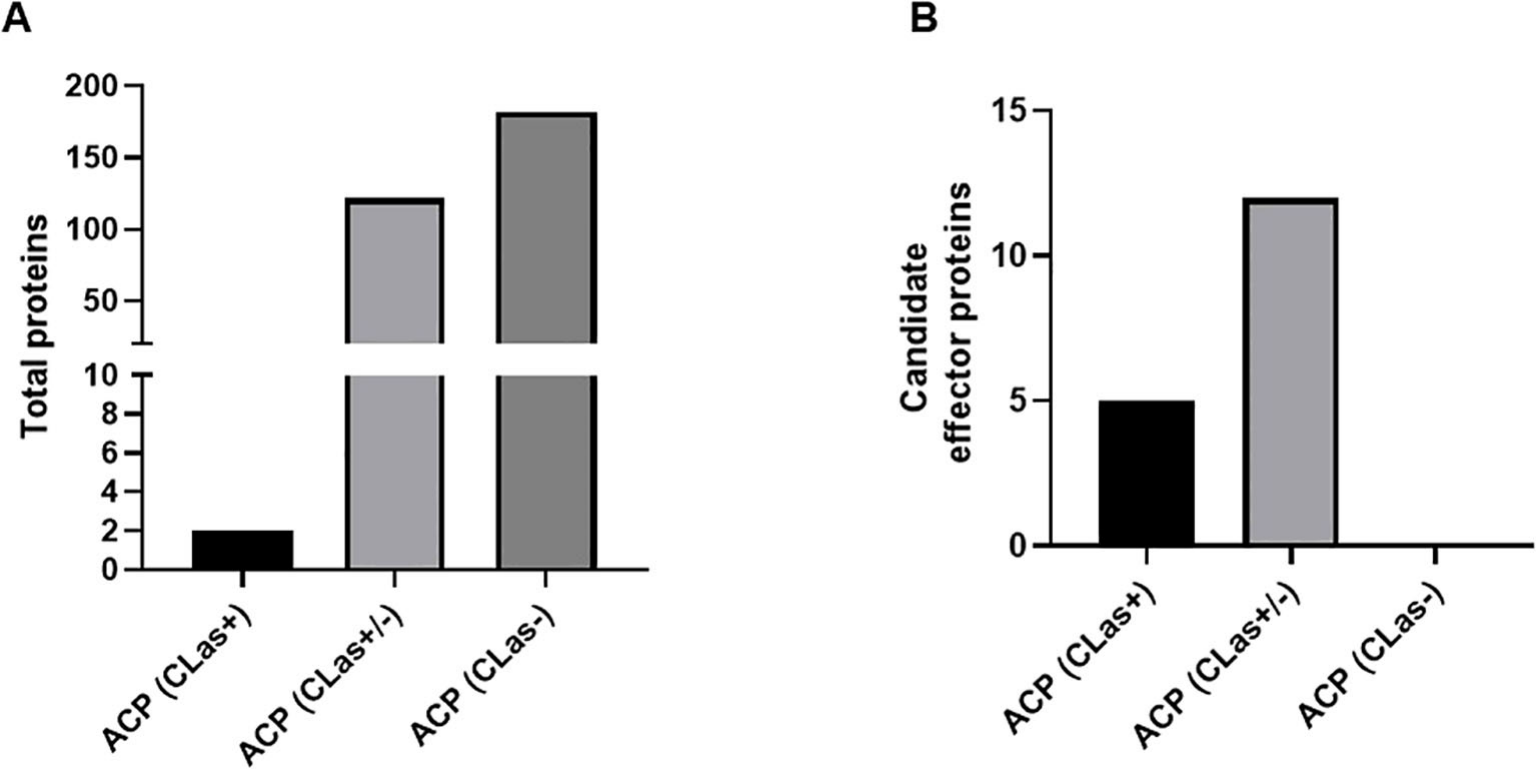
Bar plot of proteins obtained from the artificial diet. **(A)** Total number of proteins in artificial diet from healthy *D*. *citri* (ACP *CLas* −), both healthy and *C*Las-infected *D*. *citri* (ACP *CLas* +/−), or *C*Las-infected *D*. *citri* (ACP *C*Las+). **(B)** Candidate effector proteins obtained.

To identify potential effector proteins secreted by *D. citri*, healthy *D. citri* and *C*Las-infected *D. citri* were allowed to feed on an artificial diet. After 48h, the liquid diet was collected and analyzed for secreted proteins by mass spectrometry. Overall, we identified 306 proteins to be secreted into the artificial diet. We detected 182 specifically in the diet of healthy *D. citri* and 2 in the diet of *C*Las-infected *D. citri*, while the remaining 122 were found in both (**Figure 2A**, **Supplementary Table 3**). In total, 17 proteins were identified with a signal peptide and without a TM domain in artificial diet fed on to healthy or *C*Las-infected *D. citri*: putative defense protein Hdd11, laccase-4-like, laccase-24-like, ervatamin-C-like, chitinase-like protein EN03, 5NUC, spermine-oxidase, carbonic anhydrase, and ferritin, as well as eight uncharacterized proteins (**Figure 2B**, **Supplementary Table 3**). Considering these numbers, it becomes clear that host proteins secreted into the artificial diet by *D. citri* have no predicted signal peptide, indicating that other secretory pathways must be active in their salivary glands. For instance, we identified a laccase among the 306 proteins, and it does not contain any signal peptide. However, in whitefly, it has been reported to act as an effector protein (Yang et al., 2017). Laccase helps to overcome the chemical defense of plants and facilitates feeding. Thus, with our criteria for predicting effectors from the RNA-seq data, we are only looking at a small subset of the secreted proteins. Furthermore, of the 17 proteins, 5 were found in the saliva of healthy psyllids and 12 in the saliva of both *C*Las-infected and healthy *D. citri*. No proteins with SP and without TM domain were identified that were specific to *C*Las-infected psyllids only (**Figure 2B**).

### Identification of putative effector proteins from phloem exudates of host plants

As *D. citri* feed on phloem sap of host plants and secrete salivary proteins, including effector proteins, and *C*Las into phloem of host plants, we set out to detect these proteins in phloem exudates. *C*Las multiplies inside the host’s phloem sap, as it contains all the essential nutrients needed for its growth. Here, we used four genotypes: ‘Valencia’ sweet orange, *M. paniculata* (orange jasmine), *B. koenigii* (curry leaves), and *E. glauca* (Australian desert lime) to identify potentially effector proteins secreted by psyllids and *C*Las. All genotypes under study are susceptible to *D. citri*; however, low nymph viability has been observed on *E. glauca* (Damsteegt et al., 2010; Beloti et al., 2017; Tomaseto et al., 2019; Eduardo et al., 2022). In addition, Valencia is a susceptible host for *C*Las, *B. koenigii* and *E. glauca* are resistant, whereas *M. paniculata* is a transient host (Beloti et al., 2018; Alves et al., 2021, 2022, 2025).

Healthy *D. citri* and *C*Las-infected *D. citri* were confined on V3 flush shoots of the four genotypes for 1, 6, and 30 dpi. LC-MS/MS analysis of phloem exudates collected at different time points resulted in the identification of 2,214 plant proteins, 179 *D. citri* proteins, and 24 *C*Las proteins (**Supplementary Table 4**). Among the 24 *C*Las proteins detected in phloem exudates, we observed 14 proteins that, while not fitting this classical effector profile, may still contribute to *C*Las pathogenicity or survival within the plant host. Several of these are core components of bacterial metabolism and protein synthesis, including DnaK, a chaperone involved in protein folding and stress response (Fink, 1999), which in other bacteria has been linked to immune modulation; Clp protease ATP-binding subunit, known to regulate proteostasis; and virulence factors (Queraltó et al., 2023) and elongation factor G, RNA polymerase subunits, RecA, and UvrA, which may be released via outer membrane vesicles and have immunogenic potential. Enzymes such as carbamoyl-phosphate synthase, phosphoglucosamine mutase, citrate synthase, methionine adenosyltransferase, ribose-phosphate pyrophosphokinase, and argininosuccinate lyase, although primarily metabolic, could alter host cell function or immunity upon secretion. Notably, two proteins were uncharacterized, but their consistent presence in the phloem and absence of homologs in host plants make them intriguing candidates for further functional analysis. SignalP and TMHMM analyses indicated that while some proteins contain transmembrane helices, others may follow non-classical secretion pathways. Although none matched known *C*Las effectors like SDE1 or SC2_gp095, the presence of these proteins in phloem sap supports the possibility of alternative mechanisms of host manipulation, including secretion via outer membrane vesicles.

**Figure 3:**
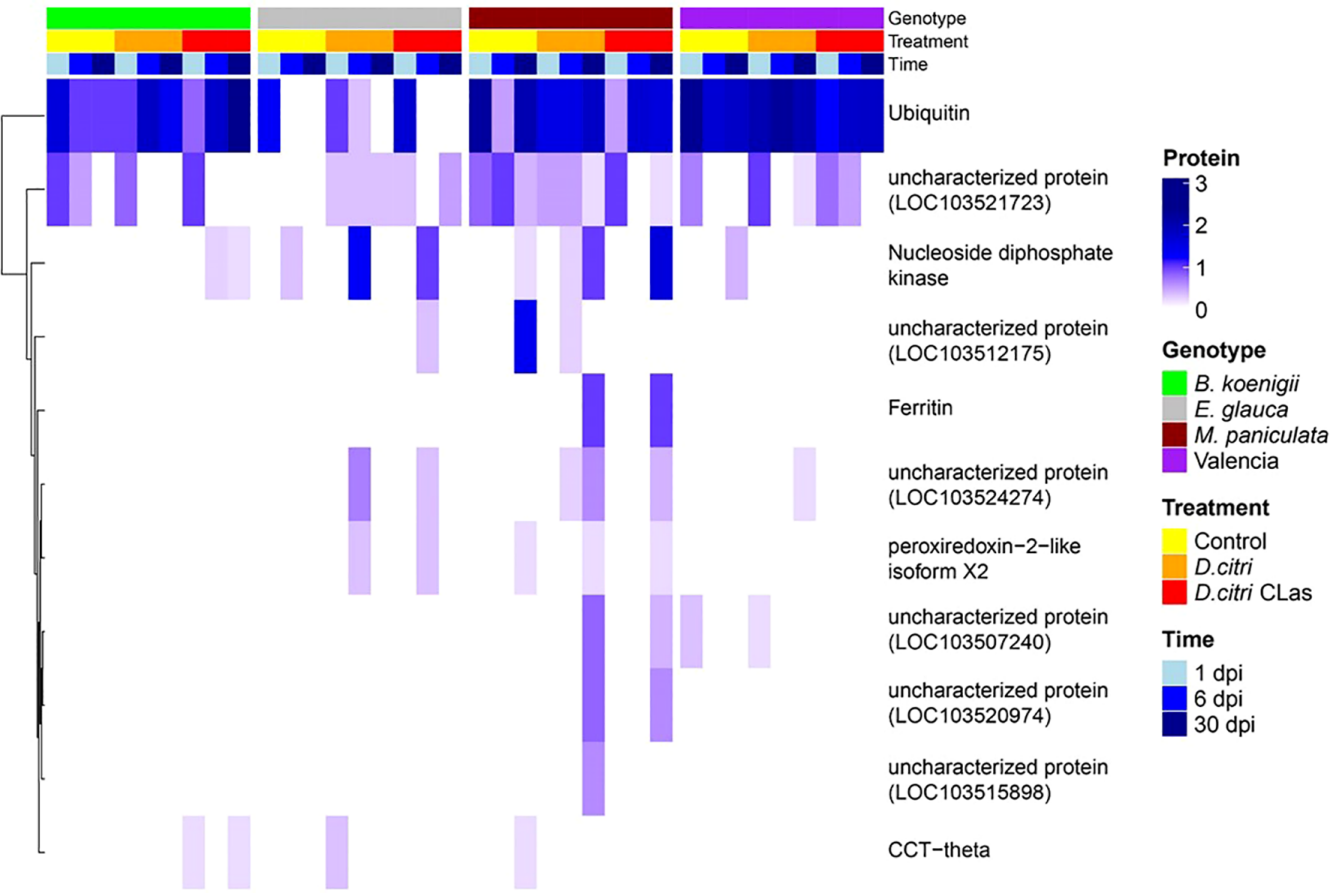
Heatmap of candidate effector proteins obtained from phloem exudates of four plant genotypes. *Bergera koenigii* (green), *Eremocitrus glauca* (gray), *Murraya paniculata* (dark cherry red), and *Citrus sinensis* (Valencia, purple) at 1 dpi (sky blue), 6 dpi (blue), and 30 dpi (dark blue). Treatments are control (yellow), *D. citri* without *C*Las (orange), and *D. citri* with *C*Las (red). The gradient in dark blue color represents the number of proteins obtained in phloem exudates.

Of the 179 *D. citri* proteins, 10 had a signal peptide and no transmembrane domain (**Supplementary Table 5**). These 10 proteins include nucleoside diphosphate kinase, peroxiredoxin-2-like isoform X2, ferritin, T-complex protein 1 subunit theta (CCT-theta), and six uncharacterized proteins. Nucleoside diphosphate kinase and the uncharacterized protein LOC103521723 were found in all four host genotypes. CCT-theta was detected in *B. koenigii*, *E. glauca*, and *M paniculata*. Peroxiredoxin-2-like isoform X2 was detected in *E. glauca* and *M. paniculata*, whereas ferritin was only identified in *M. paniculata* (**Figure 3**, **Supplementary Table 5**). No putative *C*Las effector proteins were detected in any of the phloem samples.

For functional analysis, we chose ferritin as it was present in the proteomics analysis of both artificial diet and phloem exudates. It showed significant abundance across all time points, and it has been reported in whitefly (BtFer1) to suppress host plant defense (Su et al., 2019). Thus, in total, four *D. citri* effector proteins were chosen for functional analysis.

### Proteomics analysis of phloem exudates

**Figure 4:**
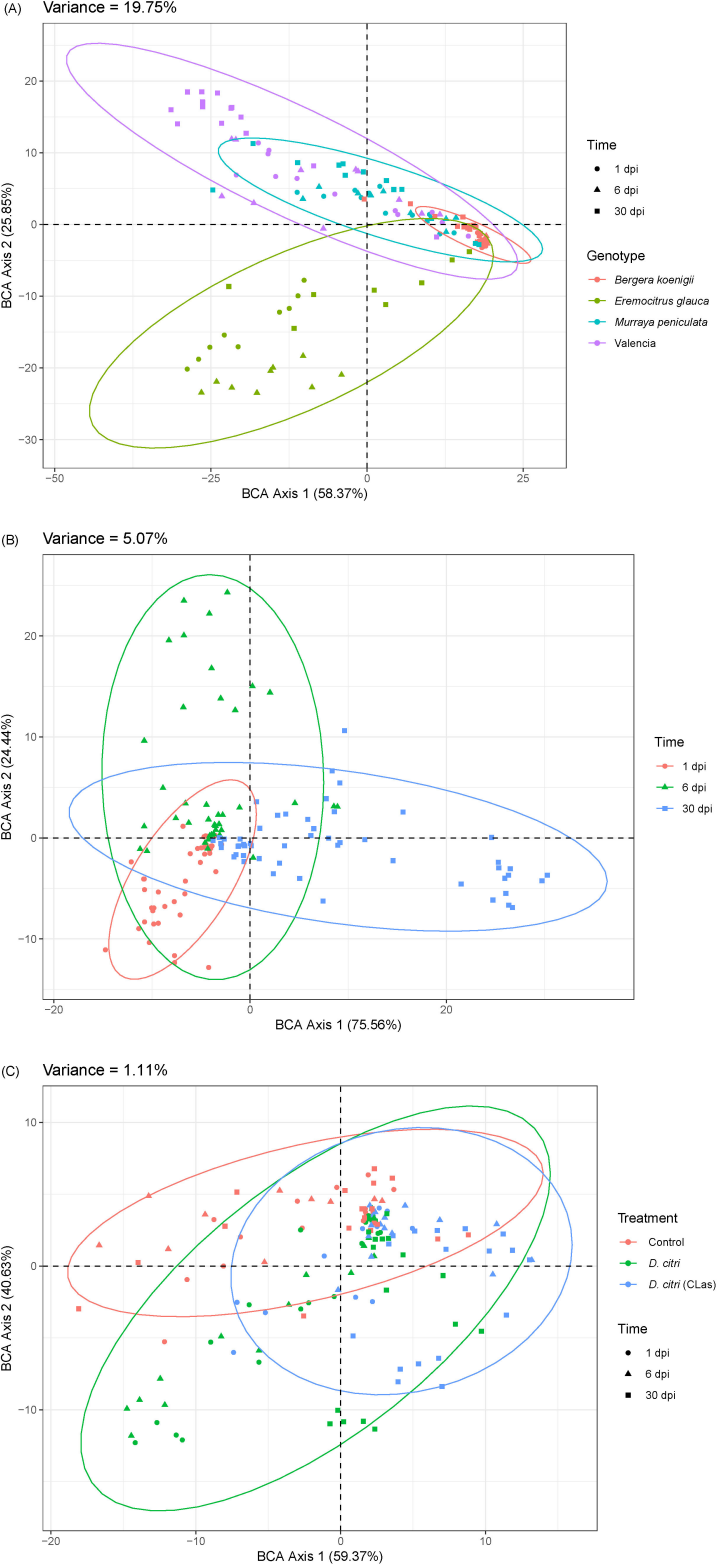
Variance partitioning analysis of proteomics profile variability according to **(A)** genotype, **(B)** time after infection, and **(C)** infection treatment.

From the proteomics analysis of the phloem exudates, we could also deduce that the four plant genotypes responded to the presence of healthy *D. citri* and *C*Las-infected *D. citri* by changing their proteomics profile. First, we used a variance partitioning analysis to evaluate the major factors contributing to the proteomics variability, and this allowed us to quantify the relative importance of the genotype, time, and treatments. Genotype had the strongest effect, explaining 19.75% of the total variability (*P*<0.001, **Figure 4A**), followed by time (5.07%, *P*<0.001, **Figure 4B**), and treatment (1.11%, *P*<0.001, **Figure 4C**). This showed that the differences in the proteome profile induced by the different treatments (exposed to *C*Las-infected *D. citri*, healthy *D. citri*, or no *D. citri*) are proportionally smaller than those of the genotypes.

**Figure 5:**
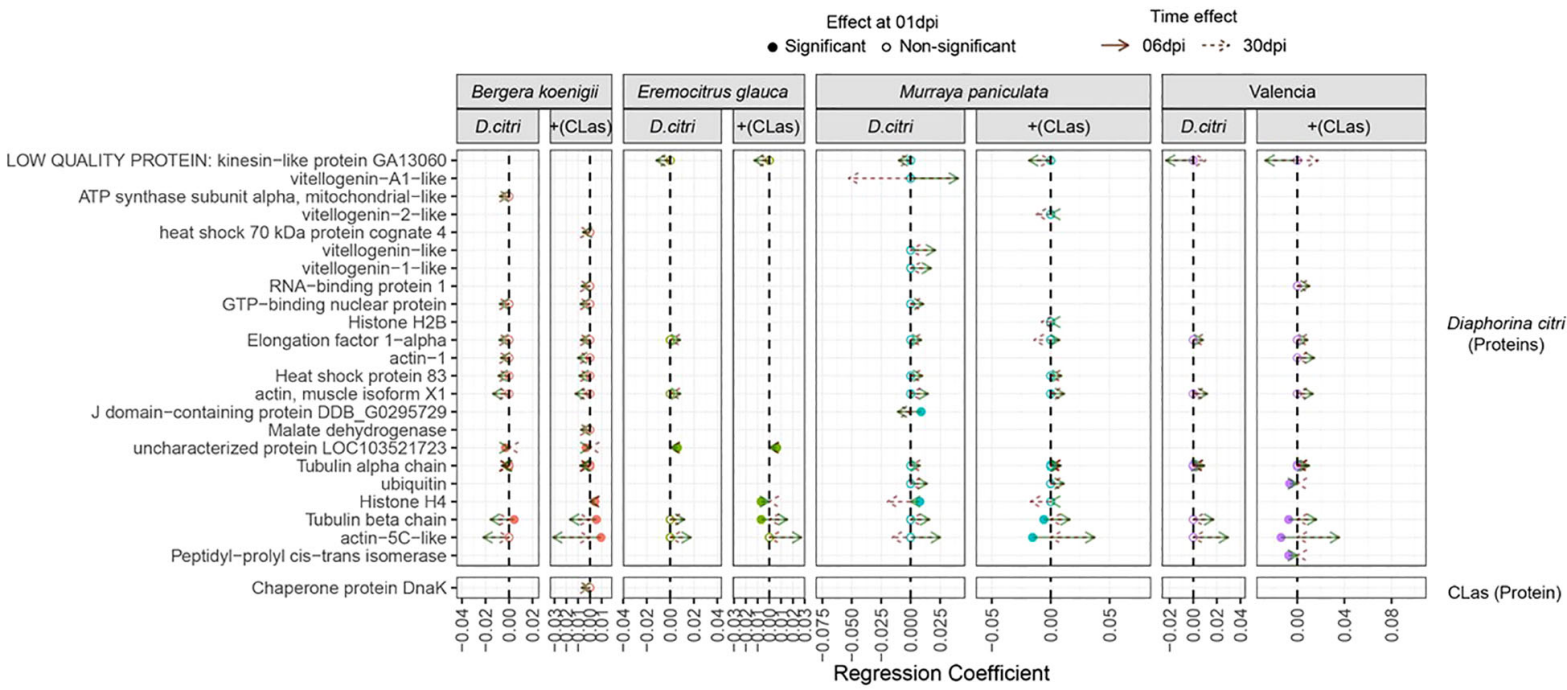
Shifts in *D. citri* and *C*Las protein levels across different genotypes of *Citrus sinensis* in response to healthy and *C*Las-infected *D. citri*. The figure compares protein expression changes in infected treatments relative to the control (non-infected). Full circles indicate proteins with significant differences at 1 dpi, while empty circles represent proteins significantly affected later (6 or 30 dpi). Green arrows denote shifts in protein expression (regression coefficients) observed at 6 dpi, and brown arrows indicate changes at 30 dpi.

A complementary model-based analysis identified 113 proteins with significantly altered levels, including 88 host-derived, 23 *D. citri* -derived, and only one from *C*Las (Chaperone protein DnaK). Protein changes followed two patterns: fast (1 dpi) and late responses (6–30 dpi) (**Figure 5**). For the genotype *B. koenigii*, we found that 16 host proteins had higher levels when infested with *D. citri*. The number increased to 21 for *C*Las-infected *D. citri* (**Supplementary Figure 1**). Interestingly, for the genotype *E. glauca*, 7 host proteins were lower when infested with *D. citri*, but when the psyllids were infected with *C*Las, the number of downregulated host proteins increased to 12. Still, we also observed that four proteins were upregulated. Similarly, for the *M. paniculata* genotype, we observed 11 proteins that are downregulated when *D. citri* were present and only five upregulated, but when *D. citri* were infected with *CLas*, the number of downregulated proteins from the plant was reduced to six and the number of upregulated proteins remained five. Finally, for the ‘Valencia’ sweet orange genotype, we report 19 proteins that were downregulated and six that were upregulated. The presence of *C*Las-infected *D. citri* resulted an increase of downregulated proteins in the host to 32 with only two proteins upregulated. In summary, there is a strong genotype-dependent response in the phloem to the *C*Las infection, and the more resistant genotypes had an increased number of citrus proteins, the levels of which were affected (**Supplementary Figure 1**).

The analysis revealed unique plant proteins with significant changes in levels under the *C*Las-infected *D. citri* treatment with a genotype- specific response (**Figure 5**). For *B. koenigii*, we observed changes in Bet v1 domain protein and fructose-bisphosphate aldolase as uniquely assigned to the treatment with *C*Las-infected *D. citri*. For *E. glauca*, we found four proteins uniquely affected when *D. citri* were infected with *C*Las (chitin-binding type 1 domain, malate dehydrogenase, fructose-bisphosphate aldolase, and sucrose synthase). For *M. paniculate*, we found one peroxidase and one ADP/ATP translocase as uniquely affected by the *C*Las-infected *D. citri* treatment. Finally, for the ‘Valencia’ sweet orange genotype, we found only one protein uniquely associated with the *C*Las-infected psyllid treatment, the Bet v1 domain protein, in the late response.

*Diaphorina citri*-derived proteins also showed genotype- and time-dependent changes. Most occurred at 6 and 30 days after infection (**Figure 5**, **Supplementary Table 6**). In *B. koenigii*, we observed a total 10 *D. citri* proteins (2 fast and 8 late), which increased to 13 (3 fast and 10 late) for the *C*Las-infected *D. citri* treatment. On the contrary, in *E. glauca*, under the healthy *D. citri* treatment, we found six proteins (one fast and five late) and one less (three fast and two late) for the *CLas*-infected psyllid treatment. A similar reduction was observed for the *M. paniculata* response to the *D. citri*: a total of 14 proteins (3 fast and 11 late) changed after 6 and 30 dpi, whereas 11 proteins (2 fast and 9 late responses) significantly changed with *C*Las-infected *D. citri*. For the most susceptible genotype ‘Valencia’ sweet orange, in total, 6 proteins (all late) were found that increased to 10 (4 fast and 6 late) when the psyllid was infected with *C*Las.

For *E. glauca*, we detected fewer *D. citri* proteins and only one uniquely presented when the genotype was subjected to a *CLas*-infected *D. citri* (histone H4 protein). For all the other genotypes, we found more uniquely associated proteins. For *B. koenigii*, we found a heat shock 70 kDa cognate protein and histone H4 uniquely affected when *D. citri* was infected with *C*Las. For *M. paniculate*, we detected a vitellgenin-2- like protein, a tubulin beta chain, and actin-5C-like proteins. For the ‘Valencia’ sweet orange, we report four proteins that changed in levels only with the *C*Las-infected *D. citri* treatment (RNA-binding protein 1, actin-1, ubiquitin, and peptidy-propyl cis-trans isomerase). In summary, there is a strong genotype-dependent response to the *C*Las infection.

### Identification of putative *C*Las effector proteins in insects using transcriptomics

To support our search for *C*Las effectors, we used the transcriptomic data from Darolt et al. (2021) generated from midguts of *C*Las-infected psyllids, the primary interface of *C*Las interacting with the psyllid vector. In total, 169 *C*Las genes were expressed exclusively in *C*Las-infected midguts of psyllids (**Supplementary Table 7**). *C*Las secretes effectors through the SEC-dependent pathway, and these Sec-delivered effectors carry an N-terminal secretion signal peptide (Sugio et al., 2011). The amino acid sequences of the 169 proteins were screened for the presence of signal peptides using SignalP 4.1 and the absence of a TM domain using TMHMM v. 2.0. Out of the 169, 10 proteins had a signal peptide and no TM domain (**Tables 1**, **2**). Seven of these were annotated as hypothetical proteins and others as flagellar basal body L-ring protein, D-alanyl-D-alanine carboxypeptidase and ABC transporter substrate binding protein. It has been reported that *C*Las secretes a minimum of 86 putative proteins via the Sec-dependent pathway (Prasad et al., 2016). Among these 86 proteins, 27 were identified as Sec-dependent effectors (SDEs). Furthermore, these SDEs have different expression levels in psyllids and citrus plants (Thapa et al., 2020). The 10 putative *C*Las effectors identified by us were aligned with these 27 SDEs. Five proteins were found to be Sec-dependent proteins: CLIBASIA_ 00460, CLIBASIA_ 00530, CLIBASIA_ 04560, CLIBASIA_ 05315, and CLIBASIA_ 05320 (**Table 2**). In addition, CLIBASIA_04320 and CLIBASIA_00530 were highly expressed in psyllids, whereas CLIBASIA_04560, CLIBASIA_05315, and CLIBASIA_05320 have been reported to have high expression in citrus (sweet orange) (Thapa et al., 2020). CLIBASIA_05315, also known as SDE1, the most studied effector, interacts and inhibits citrus papain-like cysteine protease (PLCPs) that is involved in regulating plant defenses (Clark et al., 2018). Since CLIBASIA_04560 and CLIBASIA_05320 are also expressed in the heads of *C*Las-infected *D. citri* (**Supplementary Figure 2**), both effectors were chosen for functional analysis. Their presence in the head suggests that they may be secreted via the salivary glands into the host plant during feeding, making them particularly relevant for studying host manipulation.

**Table 1:** Steps for the prediction of candidate effectors of *C*Las.

Genes from Las III phase	169
With signal peptide (SignalP 4.1)	12
Without the transmembrane domain TMHMM	10

**Table 2:** List of candidate effector proteins of *C*Las.

Number	Protein ID	Description	CLIBASIA annotation	Expression
1	WP_012778374.1	Hypothetical protein		
2	WP_012778427.1	Hypothetical protein	(CLIBASIA_00460)	High in psyllids
3	WP_015452324.1	Hypothetical protein	(CLIBASIA_00530)	High in psyllids
4	WP_012778593.1	Flagellar basal body L-ring protein FIgH		
5	WP_012778654.1	D-alanyl-D-alanine carboxypeptidase		
6	WP_015452595.1	ABC transporter substrate-binding protein		
7	WP_015824958.1	Hypothetical protein	(CLIBASIA_04560)	High in citrus
8	WP_054105251.1	Hypothetical protein		
9	WP_015452985.1	Hypothetical protein/(SDE1)	CLIBASIA_05315	High in citrus
10	WP_015452986.1	Hypothetical protein	CLIBASIA_05320	High in citrus

### Expression of *Diaphorina citri* effector proteins *in planta*

The function of proteins is closely related to their expression and location in cells. Therefore, we first determined the expression of all psyllid effector proteins in *N. benthamiana*. To investigate the subcellular localization of psyllid effectors *in planta*, the coding sequence (without signal peptide) of ferritin, prisilkin, CG31997-PA, and PCBD was fused at their N-terminus to GFP in the gateway binary vector pGWB452 under control of the 35S promoter. The expression of prisilkin, CG31997-PA, and PCBD proteins was detected after 48 hpi (hours post -*Agrobacterium* infiltration) using confocal fluorescence microscopy (**Supplementary Figure 3**). However, we could not detect GFP-ferritin in the epidermal layers of *N. benthamiana*. Still using qPCR, we detected high gene expression for *ferritin* in *N. benthamiana* (**Supplementary Figure 4**), indicating *ferritin* is indeed expressed *in planta*. This suggests that ferritin protein may not accumulate to detectable levels, or its fusion to GFP may affect its stability or proper localization.

### *Diaphorina citri* effectors modulate ROS production in *Nicotiana benthamiana* triggered by flg22

**Figure 6:**
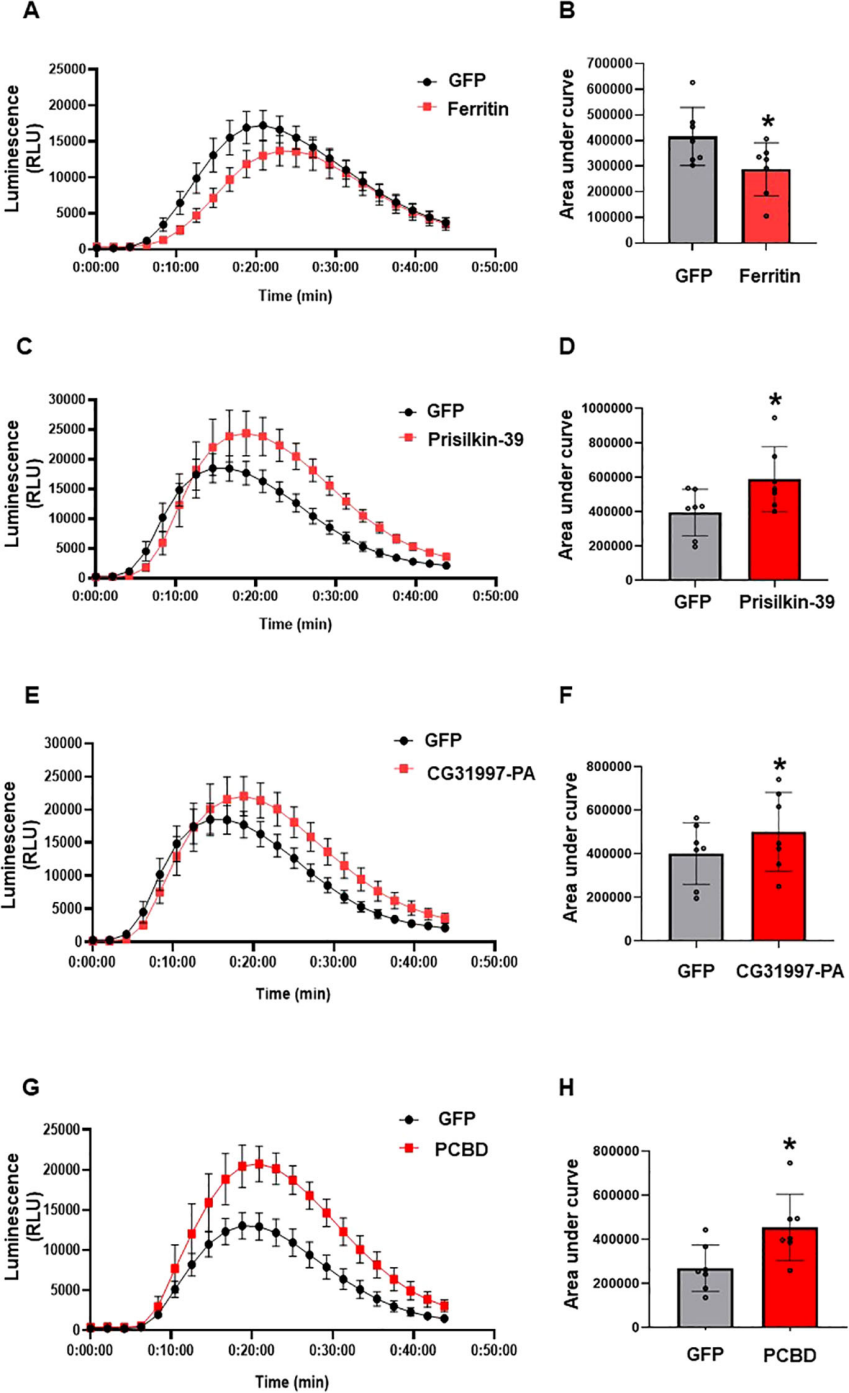
Determination of ROS modulation by ACP effectors *in planta*. Control (GFP), ferritin, prisilkin, CG31997-PA, and PCBD were transiently expressed in the epidermal layer of *N. benthamiana* leaves under the control of the 35S promoter. ROS production was measured using a luminol assay after induction with flg22 for 45min. **(A)** ROS production by ferritin and GFP. **(B)** Area under the curve of ROS produced by ferritin and GFP. **(C)** ROS production by prisilkin and GFP. **(D)** Area under the curve of ROS produced by prisilkin and GFP. **(E)** ROS production by CG32997-PA and GFP. **(F)** Area under the curve of ROS produced by CG31997-PA and GFP. **(G)** ROS production by PCBD and GFP. **(H)** Area under the curve of ROS produced by PCBD and GFP. The results are presented as mean values ± SD; leaves (*n*=8) were from different plants. Asterisks indicate statistical significance (*P*<0.05) compared to the control. Images were made using GraphPad Prism 8. Statistical significance was calculated using the Student’s *t*-test. The experiments were repeated three times with similar results.

ROS is involved in signaling, inducing immunity, and executing plant cell death. The ROS burst is one of the earliest defense signaling events in plant cells upon pathogen attack. Flagellin (flg22), the protein subunit building up the bacterial surface structure flagellum, acts as a PAMP in plants. Flg22 comprises 22 amino acid residues and is recognized by many plants, including *N. benthamiana* (Felix et al., 1999; Che et al., 2000). Here, we determined the modulation of the ROS burst by psyllid effectors after PAMP elicitor *flg22* treatment in *N. benthamiana*. ROS generation was measured using a luminol-based assay. The effector proteins were expressed under the control of the 35S promoter. 35S-driven GFP or CFP was used as negative control. *Agrobacterium*-mediated transient expression was used to express all effector proteins in *N. benthamiana*. The flg-induced ROS burst was significantly suppressed by the transient expression of ferritin compared to the control (**Figure 6A**), while the other effectors, prisilkin, CG31997-PA, and PCBD significantly enhanced the ROS burst compared to the control (**Figures 6B–D**). This shows that the selected effector proteins of psyllids modulate the flg22-triggered ROS burst.

### *Diaphorina citri* effectors enhance plant resistance to bacterial pathogen *Pseudomonas syringae*

**Figure 7:**
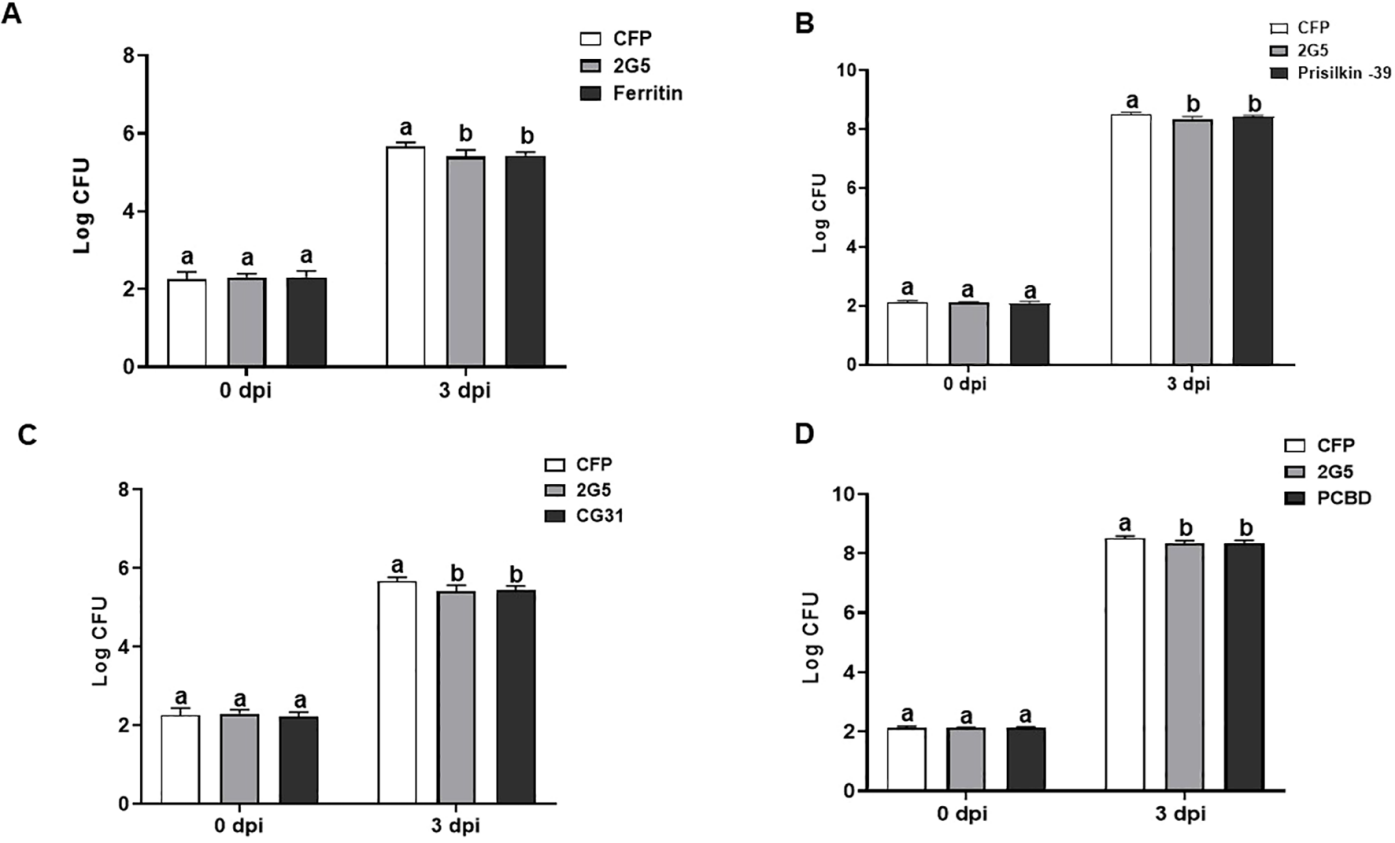
*Diaphorina citri* effectors suppress bacterial growth in *N. benthamiana*. Total population size (CFU) of *Pseudomonas syringae* pv. *tabaci* (Pta) in the leaves were measured at 0 and 3 days after Pta infiltration after the 48h expression of *D*. *citri* effectors ferritin **(A)**, prisilkin **(B)**, CG31997-PA **(C)**, and PCBD **(D)**. Bars represent the mean value ± SD (*N*=4). Infiltration with whitefly effector 2G5 and *Agrobacterium* with empty vector (EV) suspension was used as positive and negative controls, respectively. The results are presented as mean values ± SD; leaves (*n*=5) were from different plants. Different letters (a, b, and c) within a day indicate statistically significant differences (*P*<0.05). Statistical significance was calculated using one-way ANOVA (Tukey’s test). The experiments were repeated three times with similar results.

To investigate the impact of psyllid effector-modulated ROS burst on plant defense, we measured the propagation of the bacterial pathogen *Pta* in *N. benthamiana* transiently expressing the four *D. citri* effectors. The empty vector and whitefly effector 2G5 were used as negative and positive controls, respectively. The number of *Pta* colony-forming units (CFU) in *N. benthamiana* leaves pretreated with whitefly effector 2G5 was significantly lower on days 3 and 5 than in the empty vector control (Lee et al., 2018). The number of *Pta* CFU obtained from the leaves infiltrated with psyllid effectors was significantly reduced at 3 dpi (**Figure 7**). These results show that psyllid effectors enhance plant resistance to this bacterial pathogen.

### Expression of *C*Las effector proteins *in planta*

Before studying the effect of *C*Las effectors on defense responses, we first determined the expression of *C*Las effectors in plant cells. For this, we used N- terminal translational fusions with GFP with the coding region of CLIBASIA_04560 and CLIBASIA_05320 without signal peptide and driven by the 35S promoter in the gateway binary vector pGWB452. The leaf epidermal layer of infiltrated zones of each effector showed GFP expression giving a notion that both CLIBASIA_05320 and CLIBASIA_04560 were expressed in *N. benthamiana* (**Supplementary Figure 5**).

### *Candidatus* Liberibacter asiaticus effectors regulate ROS production of *Nicotiana benthamiana* triggered by flg22

**Figure 8:**
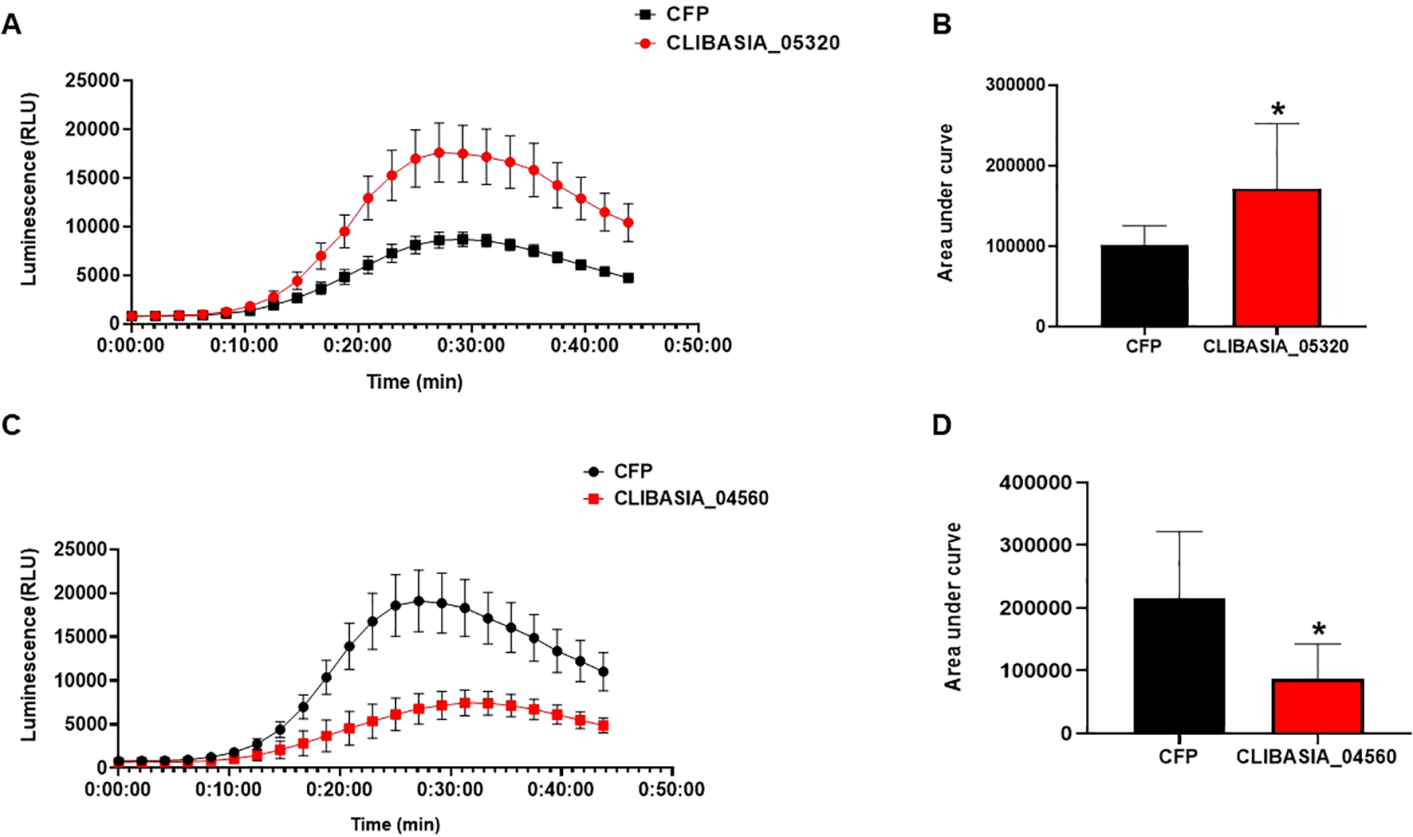
*C*Las effectors modulate ROS production *in planta*. Control (CFP), CLIBASIA_05320, and CLIBASIA_04560 were transiently expressed via agroinfiltration in the epidermal layer of *N. benthamiana* leaves under the control of 35S promoter. ROS production was measured using a luminol assay after induction with flg22 for 45min. **(A)** ROS production in *N. benthamiana* expressing CLIBASIA_05320 or CFP. **(B)** Area under the curve of ROS produced by CIBASIA_05320 and CFP. **(C)** ROS production in *N. benthamiana* expressing CLIBASIA_04560 and CFP. **(D)** Area under the curve of ROS produced by CLIBASIA_04560 and CFP. Eight plants were used for the assay. The results are presented as mean values ± SD; leaves (*n*=8) were from different plants. Asterisks indicate statistical significance (*P*<0.05). Images were made using GraphPad Prism 8. Statistical significance was calculated using the Student’s *t*-test. The experiments were repeated three times with similar results.

To functionally characterize the putative effectors, we determined their potential to modulate the ROS production induced by flg22 as described above. The infiltrated zone of *C*Las effectors, including controls, was used to measure flg22-induced ROS burst using luminol-based assay. The measurements of ROS production over time showed that CLIBASIA_04560 significantly suppressed the ROS burst compared to control, whereas CLIBASIA_05320 significantly enhanced ROS production compared to control CFP in *N. benthamiana* (**Figure 8**). This result suggests that effector proteins of *C*Las modulate PAMP-triggered ROS bursts in *N. benthamiana*.

## Discussion

HLB, also called citrus greening disease, is difficult to manage due to the non-specific nature of disease symptoms, the prolonged latency of disease in field trees, the probably non-uniform distribution of the pathogen in trees, the influence of environment (especially temperature) on symptom expression and bacterial multiplication, and the fastidious nature of the bacterium. Numerous research studies have been conducted, including the identification of the causal agent (Jagoueix et al., 1994), cellular changes in infected trees (Folimonova and Achor, 2010), and detection and confirmation of the causal agent (Li et al., 2006; Gopal et al., 2009; Nageswara-Rao et al., 2013; Aksenov et al., 2014). Despite these studies, the virulence mechanism remains largely unknown. Identifying effector proteins and their function in host plant cells and their plant target is key to understanding pathogen–plant interactions and management strategies.

### *Diaphorina citri* effectors modulate plant defense responses

Salivary proteins are important for feeding, aiding in lubrification, digestion, and penetration (Cherqui and Tjallingii, 2000), and they play key roles in plant–insect interactions (Dalio et al., 2017). Despite available proteomics data, only a few *D. citri* effectors and their roles have been reported (Yu and Killiny, 2018; Pacheco et al., 2020; Kuang et al., 2024; Liu et al., 2024). DcEF32 was shown to be essential for feeding and survival (Pacheco et al., 2020), and DcE1 inhibited plant cell death and PAMP-induced callose deposition (Kuang et al., 2024). DcitSGP1 and DcitSGP3, which are delivered into the plant during feeding, suppress the jasmonic acid (JA) signaling pathway, which is a key part of plant defense responses. Suppressing JA responses helps *D. citri* to feed from the phloem, thereby increasing the survival (Liu et al., 2024).

Our selection of prisilkin, CG31997-PA, and PCBD as potential candidate effectors was based on their expression level (RNA-seq), protein domains, and homology with effectors from other phloem feeders. In addition, ferritin was chosen as it was secreted in both phloem and artificial diet. Whitefly ferritin, BtFer, has been reported to suppress key plant defense responses, including ROS production, callose deposition, proteinase inhibitor activation, and JA-mediated signaling pathways (Su et al., 2019), indicating a role in facilitating insect feeding and survival. Given this, the conservation of ferritin between *D. citri* and whiteflies suggests a potential conserved virulence function, although experimental validation in psyllids is still required. PCBD is a bifunctional protein functioning both as a metabolic enzyme and a transcriptional coactivator (Mistry et al., 2021). Its high similarity (80%) with the whitefly homolog raises the possibility that it may contribute to metabolic regulation or modulating gene expression during plant–insect interaction. Further functional analysis will be needed to determine whether its role extends indeed to modulating host responses. Interestingly, no homologs were found for prisilkin and CG31997-PA in other phloem feeders, suggesting these genes may be *D. citri*-specific. While their functions remain unknown, their uniqueness raises the possibility that they are involved in species-specific adaptations, possibly targeting unique aspects of psyllid–host interactions. Functional analysis by knocking out these effectors in the psyllid or determining the proteins it targets in the plants should shed light on their roles.

In this study, for functional characterization, we transiently expressed the effectors in plants and determined various read outs. Because transient expression assays in citrus are extremely difficult, we used *N. benthamiana* as a model plant (Goodin et al., 2008). Insects trigger complex defenses including a ROS burst and hormone signaling mediated by JA, salicylic acid (SA), and ethylene (Acevedo et al., 2015; Bigeard et al., 2015). Ferritin suppressed flg22-induced ROS production, similar to BtFer1, which has been shown to suppress H_2_O_2_-generated oxidative signals in tomato (Su et al., 2019). In contrast, prisilkin, CG31997-PA, and PCBD enhanced flg22-induced ROS production. ROS can act both as a scavenging molecule and a signaling molecule. In plants attacked by biotrophic pathogens, it activates SA signaling which is preceded by oxidative bursts originating in different cellular compartments (Wrzaczek et al., 2013). In the case of basal (PTI) and induced (ETI) defense responses, it has been reported that increases in SA levels are preceded by apoplastic H_2_O_2_ bursts mediated by NADPH oxidases and extracellular peroxidases (Mackerness et al., 2001; Torres et al., 2002; Joo et al., 2005; Tsuda et al., 2008; O’Brien et al., 2012; Mammarella et al., 2014). In addition, it has been reported that the induced SA pathways in tobacco and *Arabidopsis* were beneficial to whitefly performance (Zarate et al., 2007; Alon et al., 2013). It is thus possible that the induced ROS by *D. citri* effectors activate SA signaling pathway that might be advantageous to *D. citri* performance. Ferritin might act later to suppress ROS production as high ROS levels are detrimental to the psyllid.

In addition, we further evaluated whether the four candidate effectors would elicit plant immunity in *N. benthamiana* against *Pta*. The full-length *D. citri* effectors that we identified in the present study reduced the bacterial growth of virulent *Pta*. These results show that *D. citri* effectors indeed enhance plant resistance to virulent bacterial pathogen in *N. benthamiana*. A previous study showed that whitefly infestation in plants induced SA responses (Park and Ryu, 2014). Further studies found that the effector proteins 2G5, 2G4, and 6A10 from *B. tabaci* (MED) and S1, B1, and P1 from *B. tabaci* (MEAMI1) induced SA-responsive genes and also reduced the bacterial growth of virulent *Pta*, both locally and systemically in *N. benthamiana* (Lee et al., 2018; van Kleeff et al., 2024). The molecular changes in citrus after *D. citri* infestation still needs to be studied in detail; however, it has been reported that the expression of *D. citri* effectors DcitSGP1 and DcitSGP3 in citrus plants suppressed JA-responsive genes favoring *D. citri* feeding (Liu et al., 2024). Thus, it can be assumed that the four *D. citri* effectors in our study induce the SA pathway, which might favor *D. citri* survival on citrus plants thereby presumably increasing the transfer of *C*Las.

### *C*Las effectors manipulate plant defense responses

Three ‘*Ca. Liberibacter*’ species, *C*Las, *C*lam (*Candidatus* Liberibacter americanus), and *C*Laf (*Candidatus* Liberibacter africanus), have been found to be associated with HLB (Bové, 2006), with *C*Las being the most widespread and the most damaging (Gottwald et al., 2007; Hodges et al., 2015). ‘*Ca. Liberibacter*’ species possess a reduced genome size of c.1.2 Mb (Leonard et al., 2012; Lai et al., 2016). *C*Las is an intracellular bacterium totally dependent on the nutritional compounds present on the phloem of the plant host to survive (Lai et al., 2016; Alves et al., 2025). Despite their lack of type III secretion systems (Duan et al., 2009), it has been proposed that *C*Las uses its complete general secretory pathway (GSP/Sec-translocon) to secrete effectors (Prasad et al., 2016).

CLIBASIA_05321, CLIBASIA_04560, and CLIBASIA_05320 were found to have higher expression levels in plants (Thapa et al., 2020). Among these three effectors, CLIBASIA_05321, also known as SDE1, has been reported to directly interact with citrus papain-like cysteine proteases to inhibit their activity and suppress plant immunity (Clark et al., 2018). Since CLIBASIA_04560 and CLIBASIA_05320 were predicted as effector candidates from our analyses, these were further taken for functional analysis. As *C*Las cannot be cultured *in vitro*, expression of the putative effectors *in planta* was needed for further characterization. It has been reported that *C*Las can multiply in tobacco (*N. tabacum* cv. Xanthi) and causes symptoms (Francischini et al., 2007); thus, it is possible that the virulence factors target a similar pathway in tobacco as in citrus. Conversely, *N. benthamiana* was used to identify a virulence factor of *C*Las that induced cell death by transient expression (Pitino et al., 2016). Hence, *N. benthamiana*, a model plant for plant–pathogen interaction (Goodin et al., 2008), was used to screen for putative effectors of *C*Las via agroinfiltration.

Plants rely on surface-localized receptors to recognize pathogens, including insects, that are activated upon insect feeding and salivary secretions in the apoplast. Phloem defense responses include the production of ROS, Ca^2+^ influx, callose deposition, and activation of proteins capable of occluding sieve elements (Huang et al., 2020). However, a detailed mechanistic understanding of phloem-mediated defense remains elusive. The putative effector CLIBASIA_04560 suppressed the flg22-induced ROS production in *N. benthamiana*. Similar results were seen for ‘*Ca. L. solanacearum’* effectors HPE2, HPE16, and HPE19. HPE2 and HPE16 suppressed chitin-induced ROS production, whereas HPE19 suppressed the flg22-induced ROS burst (Reyes Caldas et al., 2022).

Interestingly, CLIBASIA_05320 enhanced ROS production *in planta*. However, it is known that not all effectors from pathogens suppress ROS production upon the perception of pathogen feature. Indeed, most *C*Las effectors were identified to suppress defense response, but several *C*Las effectors like SDE1, AGH17470, and CLIBASIA_04425 are HR elicitors (Pitino et al., 2016; Zhang et al., 2020; Du et al., 2022; Oh et al., 2022; Zhang et al., 2023). In addition, CLIBASIA_04425 was reported to enhance ROS burst and trigger cell death upon transient expression in *N. benthamiana* (Zhang et al., 2023). It has been shown for ‘*Ca. L. solanacearum*’ that, among 19 effectors, three consistently suppressed and two enhanced ROS production. ‘ *Ca. L. solanacearum’* effectors HPE1 and HPE21 enhanced ROS production (Reyes Caldas et al., 2022). HPE1 has been reported as a variable BAX-induced cell death and Prf hypersensitive response suppressor (Levy et al., 2019). Plants infected with ‘*Ca. L*. *solanacearum*’ usually produce high amounts of ROS and other defense-related compounds (Kumar et al., 2017; Wallis et al., 2012); the fact that not all effectors suppress ROS production upon perception of pathogen features could partially explain the high accumulation of these compounds.

### Potential of proteomics of phloem exudates

One of the main questions remains where the effectors of the psyllids and bacteria exert their function. One hypothesis is that this indeed occurs in the phloem or in the phloem companion cells or perhaps the effectors move even further to other cells as is the case for effectors of phytoplasma (Sugio et al., 2011). We have taken a first step to understand this process better by isolating phloem exudates on which we performed proteomics. We did not detect a great number of bacterial proteins, and this may not be surprising considering that the overall protein composition of phloem exudates is likely dominated by plant-derived proteins, followed by insect salivary proteins, with bacterial proteins comprising only a minor fraction—especially in the absence of a systemic or severe infection. Since our approach relied on phloem exudates from *D. citri* -infested plants, the relatively low abundance of *C*Las proteins may reflect the limited proportion of bacterial proteins present in insect saliva, or the spatial localization of bacterial effectors to plant tissues beyond the phloem. In contrast, we detected a substantial number of *D. citri* -derived proteins, including both general metabolic proteins and potential effectors.

The most interesting finding is that the levels of certain plant proteins can change considerably upon *D. citri* infestation or *C*Las infection. The genotype of the plant seems to be the major factor in this. Seemingly, a genotype more resistant to *D. citri* would have less *D. citri* proteins in its phloem. Or, when considering the time component, it would change some of its protein levels more quickly upon infestation. Such a resistant genotype should also have fewer *D. citri* proteins in its phloem, and this is precisely what we saw. The number of *C*Las proteins was too limited to produce a hypothesis, but such a scenario seems plausible. Still, the questions remain: what is the cause, and what is the consequence? We also observed substantial changes in *D. citri* proteins in response to the genotypes, infection type, and time after infection. Most of these changes were evident at 6 and 30 days post-infection. These findings emphasize the reciprocal nature of plant–insect interactions, where the host plant’s proteomic responses can influence the proteins expressed by the insect vector.

In any case, the phloem seems to be a very interesting source of plant proteins that may play a role in susceptibility or resistance to *D. citri*. For instance, in whiteflies, some plant proteins with which whitefly effectors interact are also present in the phloem and might act to enhance susceptibility (Naalden et al., 2024). Thus, this is certainly worthwhile investigating further, as well as the specialized metabolites in the phloem. They might have defense functions as the most specialized metabolites. For this, more resistant genotypes are necessary (Alves et al., 2022).

In conclusion, we took an integrated approach to detect proteins secreted by *D. citri* and *C*Las. In addition to the classical bioinformatics-driven effector-mining strategy and proteomics on the saliva of *D. citri* feeding from an artificial diet, we performed proteomics on phloem exudates of trees fed upon by *D. citri*. Our findings provide insight into how *C*Las and its insect vector deploy effector proteins to manipulate host plant defenses. Since we identified effector proteins using a bioinformatics-driven effector-mining strategy focusing only on proteins with a signal peptide and no transmembrane domain, we might have missed important secretory proteins. Further work is required to functionally characterize additional effectors, especially those lacking classical secretion signals, and to validate findings in citrus. Understanding *D. citri* effectors and their role in citrus, specifically the citrus proteins with which they interact, can help in designing breeding strategies to obtain more resistant plants.

## Data Availability

The datasets presented in this study can be found in online repositories. The names of the repository/repositories and accession number(s) can be found in the article/**Supplementary Material**.
