# Supplementary material for: Identification and functional analysis of putative effector proteins from Diaphorina citri and ‘Candidatus Liberibacter asiaticus’
Source: Front Plant Sci. 2025 Nov 27;16:1656652. doi: 10.3389/fpls.2025.1656652 (PMC12695838; doi:10.3389/fpls.2025.1656652)
Supplement: Supplementary file 1 [file Presentation1.pptx]

## Slide 1
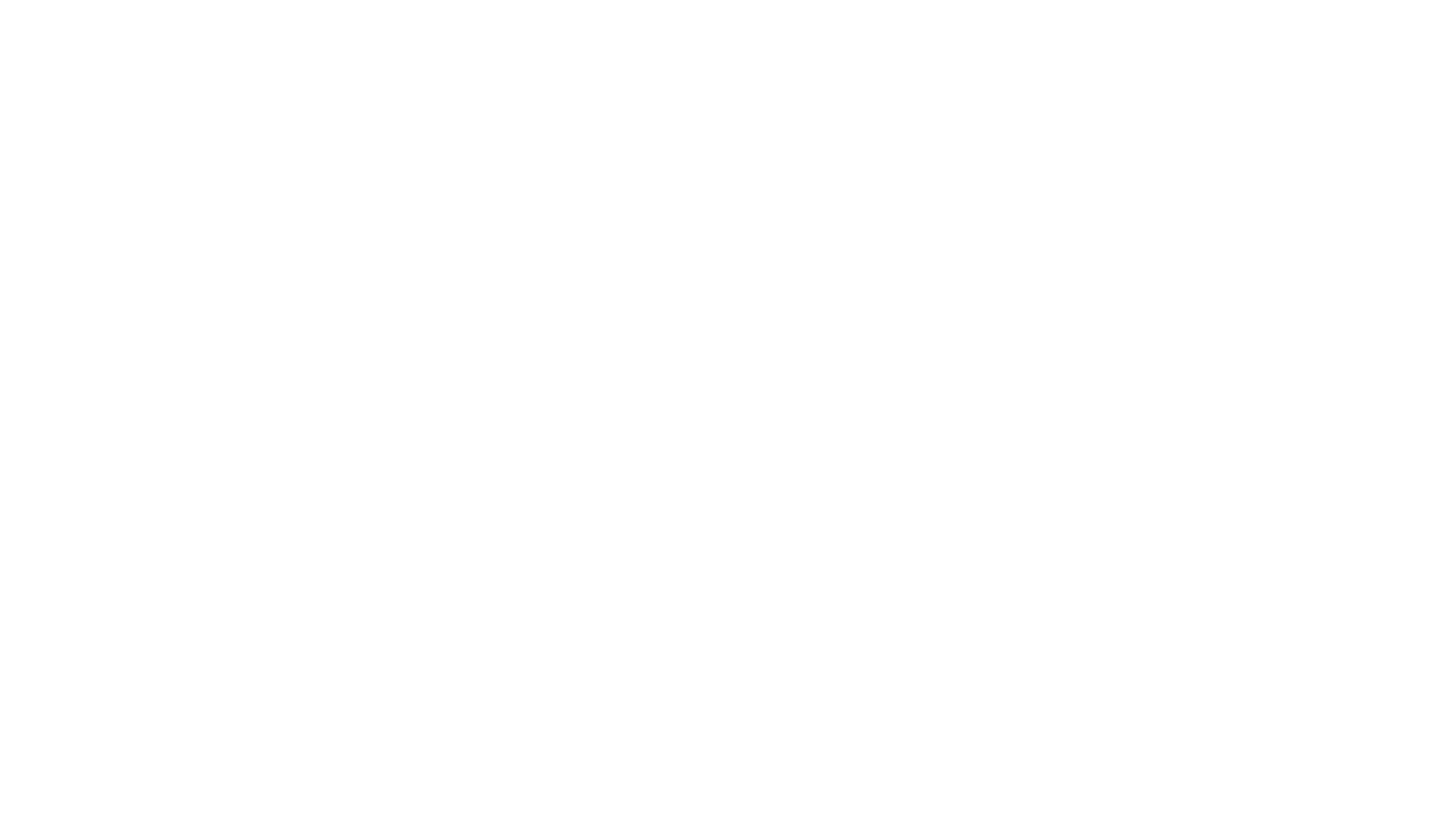

## Slide 2
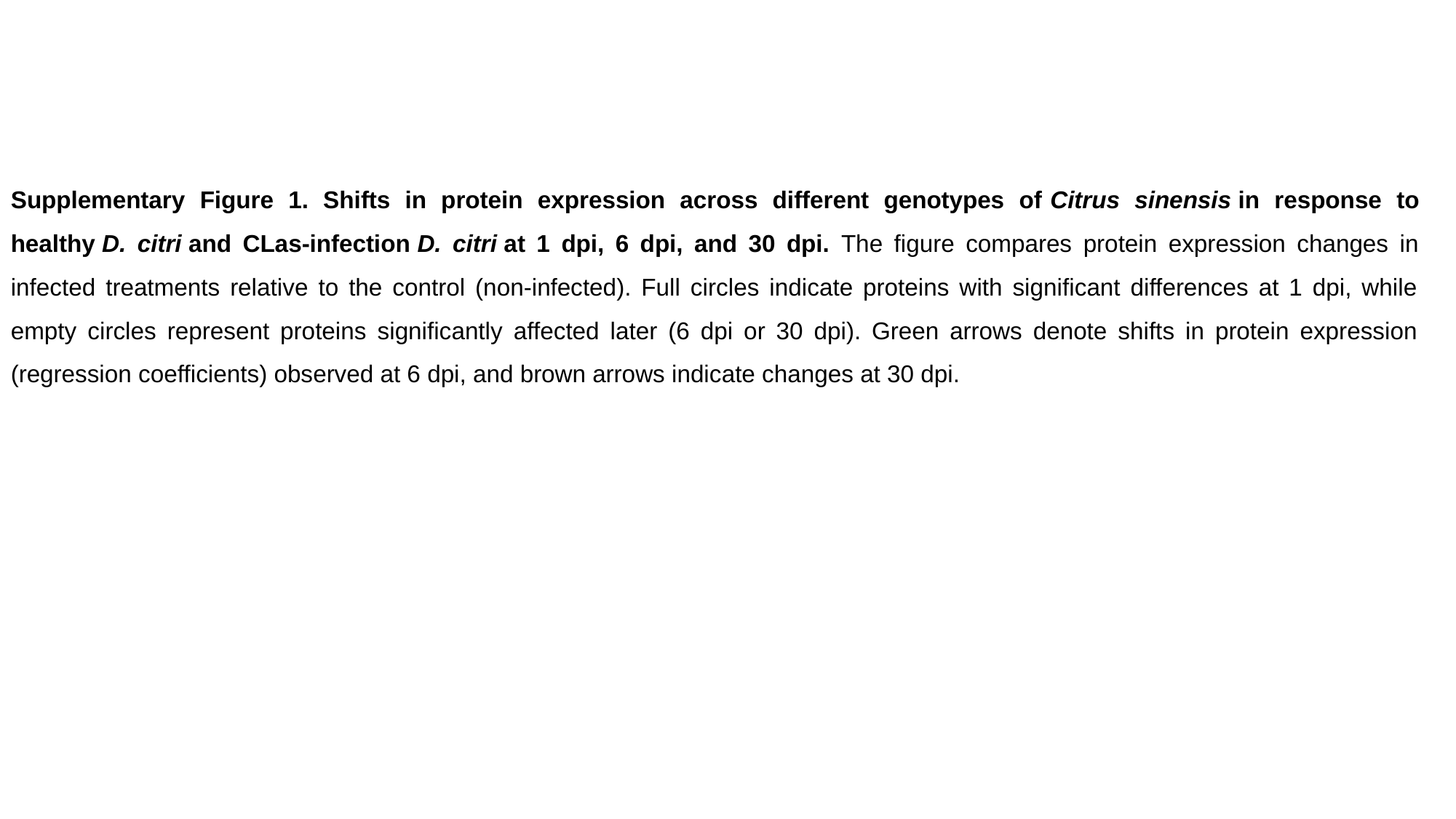

Supplementary Figure 1. Shifts in protein expression across different genotypes of Citrus sinensis in response to healthy D. citri and CLas-infection D. citri at 1 dpi, 6 dpi, and 30 dpi. The figure compares protein expression changes in infected treatments relative to the control (non-infected). Full circles indicate proteins with significant differences at 1 dpi, while empty circles represent proteins significantly affected later (6 dpi or 30 dpi). Green arrows denote shifts in protein expression (regression coefficients) observed at 6 dpi, and brown arrows indicate changes at 30 dpi.

## Slide 3
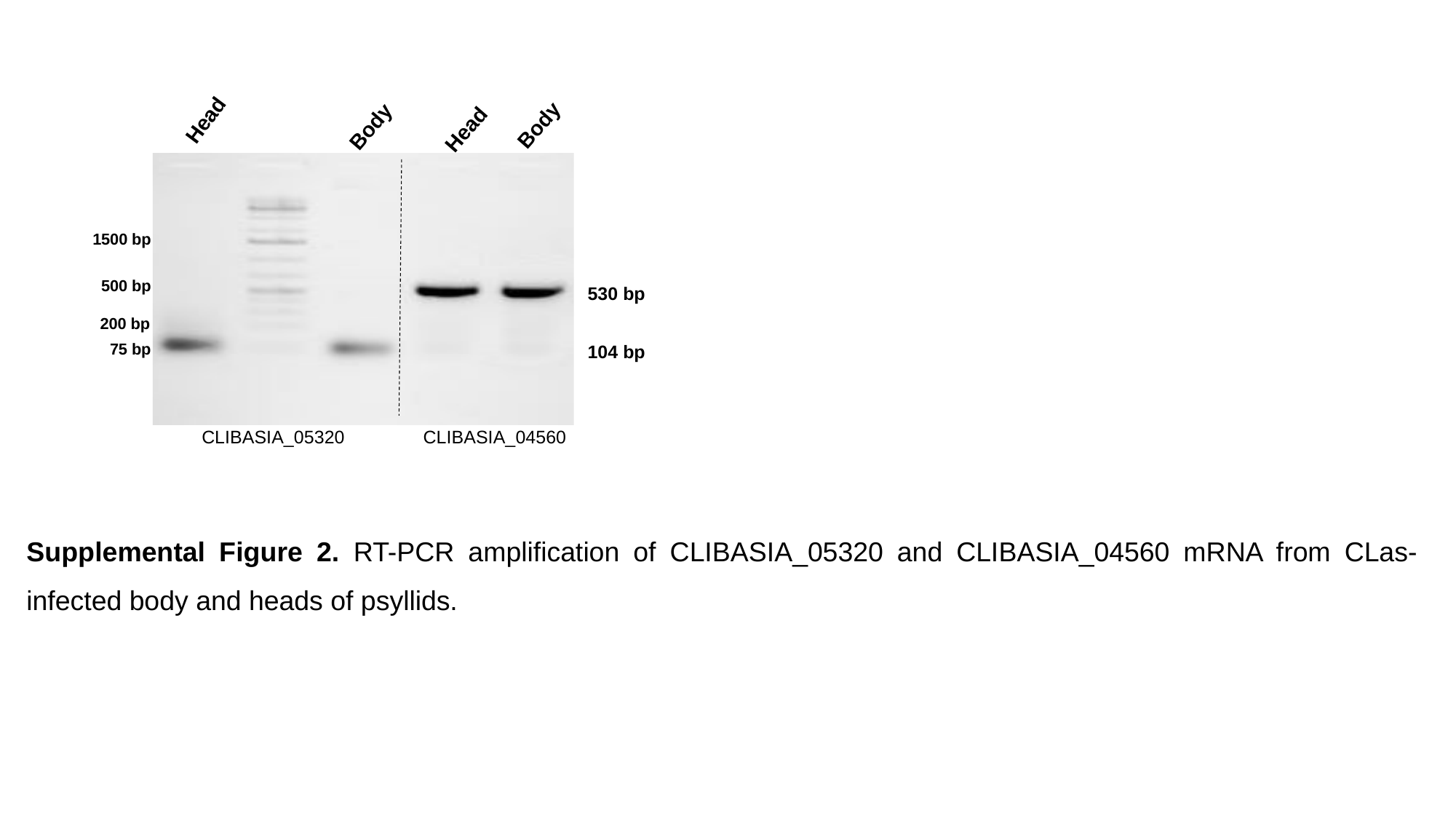

Head
Body
Body
Head
1500 bp
500 bp
200 bp
75 bp
CLIBASIA_05320
CLIBASIA_04560
530 bp
104 bp
Supplemental Figure 2. RT-PCR amplification of CLIBASIA_05320 and CLIBASIA_04560 mRNA from CLas-infected body and heads of psyllids.

## Slide 4
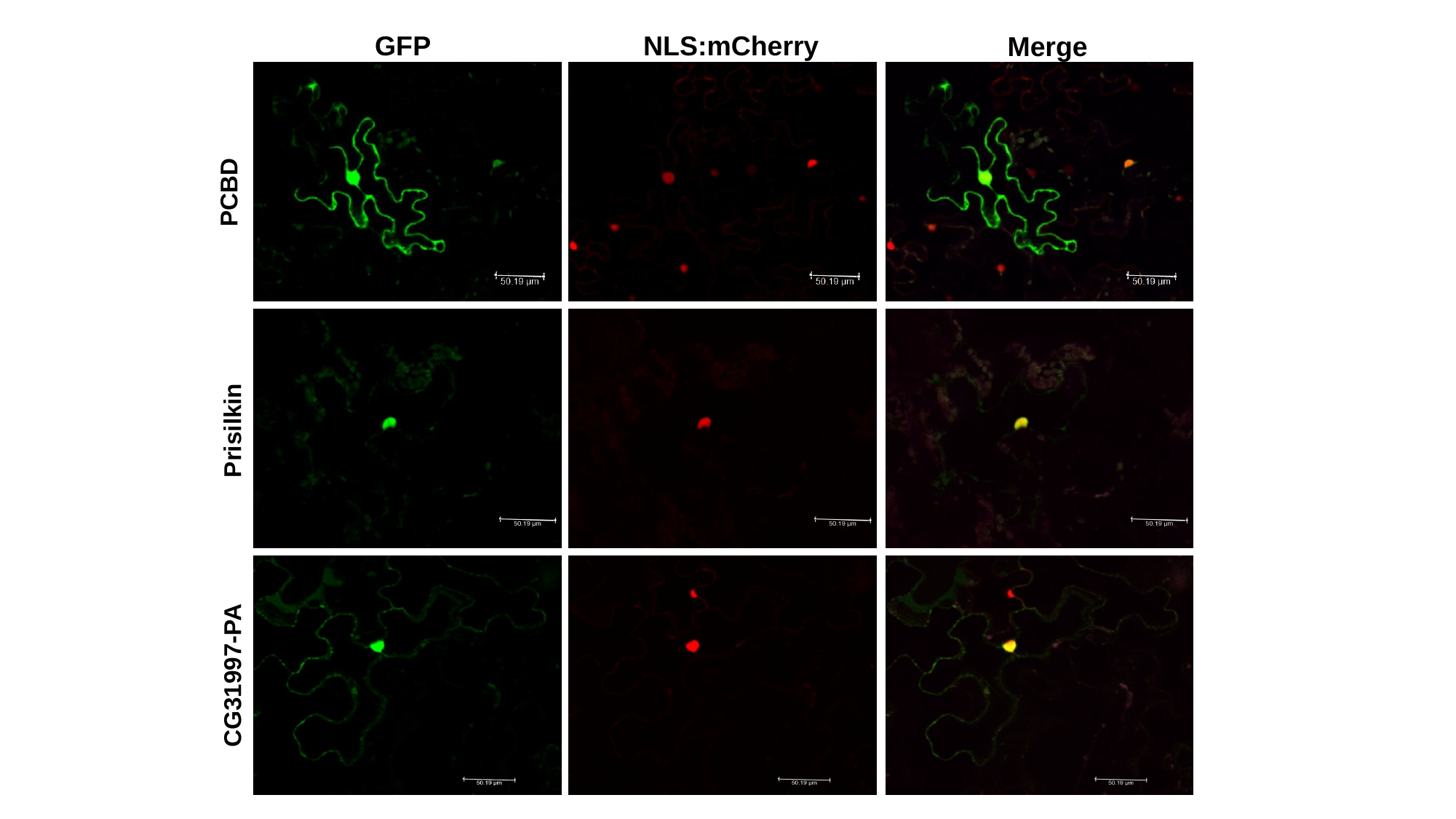

GFP
NLS:mCherry
Merge
PCBD
Prisilkin
CG31997-PA

## Slide 5
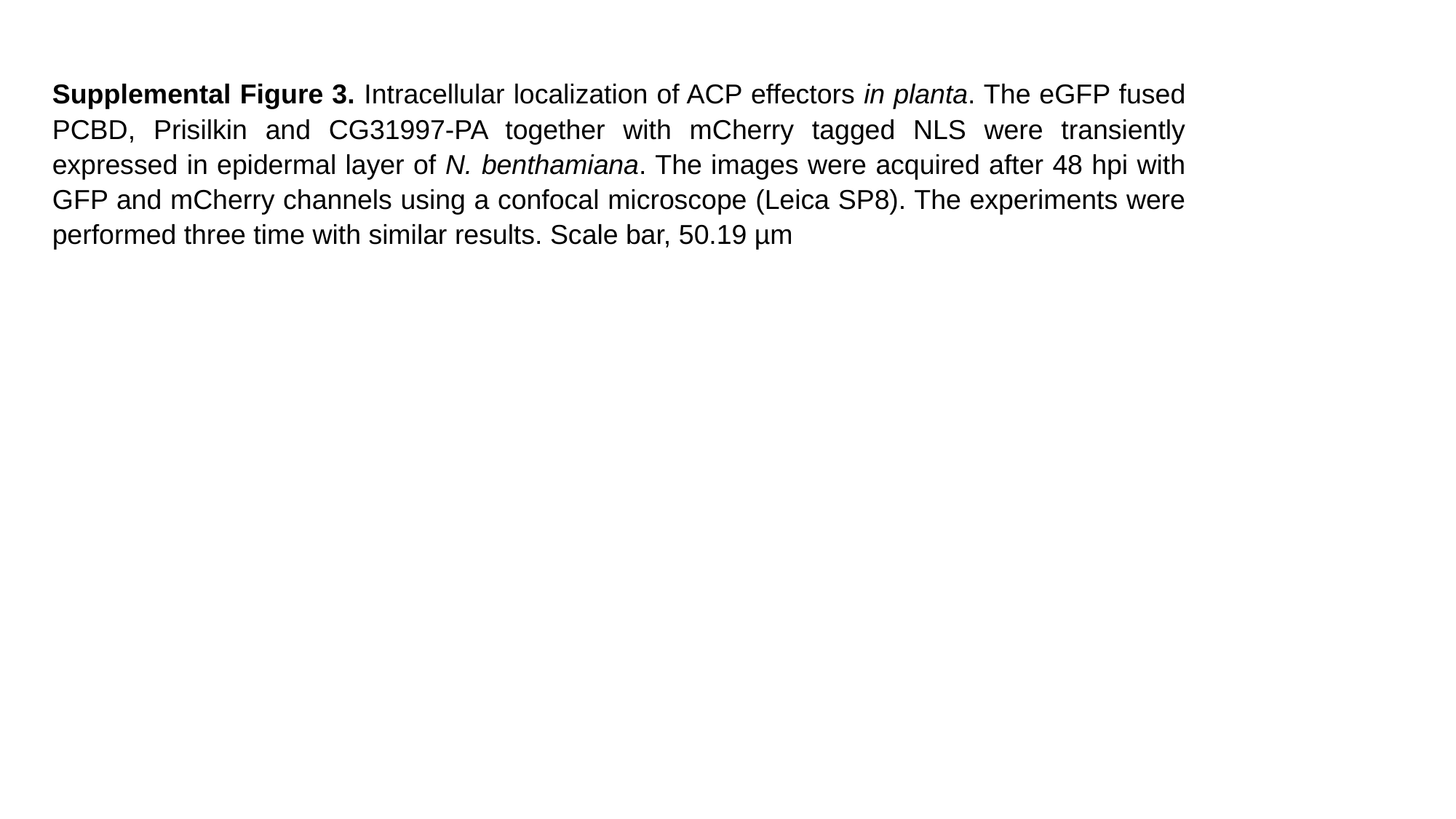

Supplemental Figure 3. Intracellular localization of ACP effectors in planta. The eGFP fused PCBD, Prisilkin and CG31997-PA together with mCherry tagged NLS were transiently expressed in epidermal layer of N. benthamiana. The images were acquired after 48 hpi with GFP and mCherry channels using a confocal microscope (Leica SP8). The experiments were performed three time with similar results. Scale bar, 50.19 µm

## Slide 6
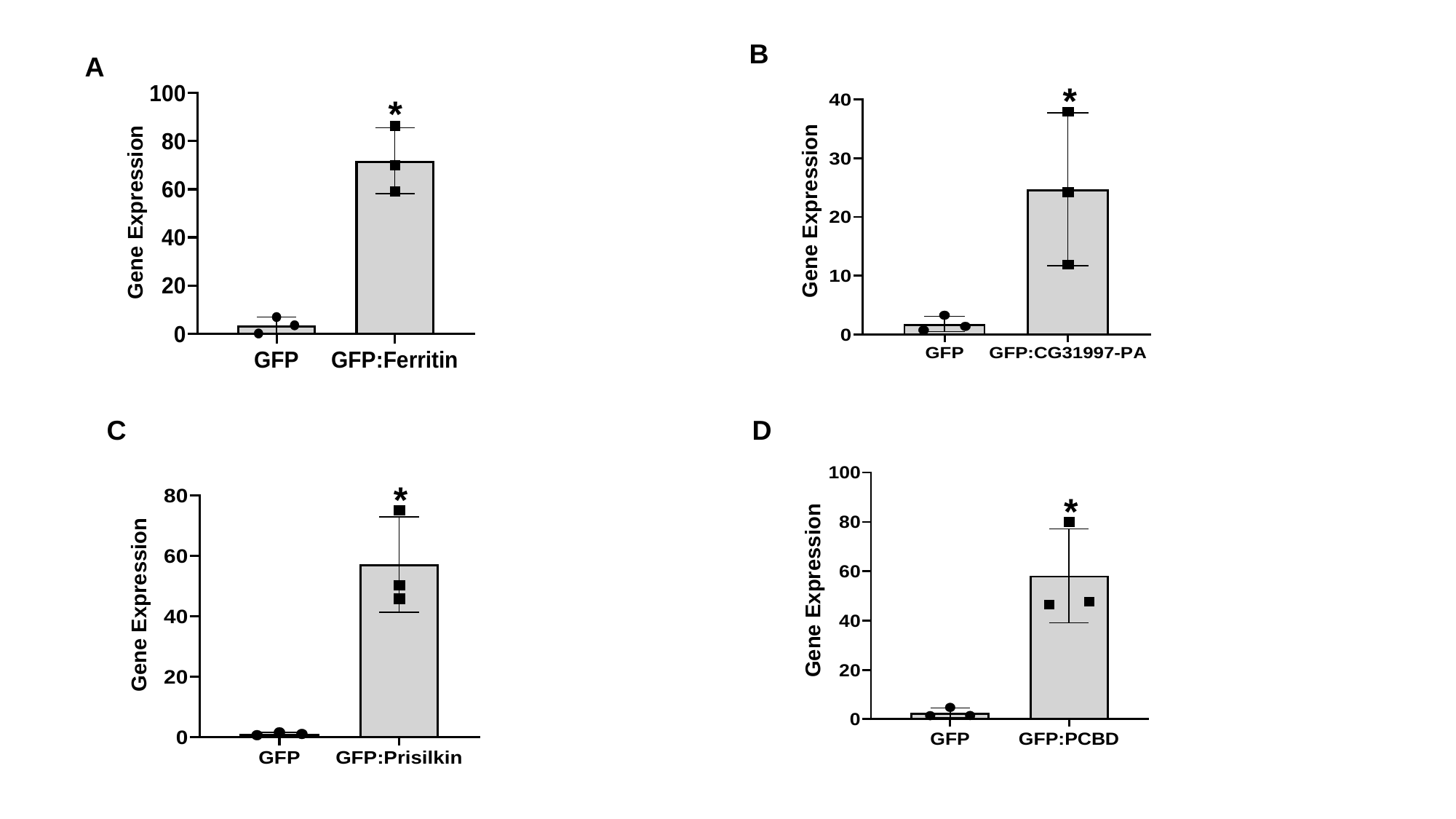

B
A
Gene Expression
*
Gene Expression
*
C
D
Gene Expression
*
Gene Expression
*

## Slide 7
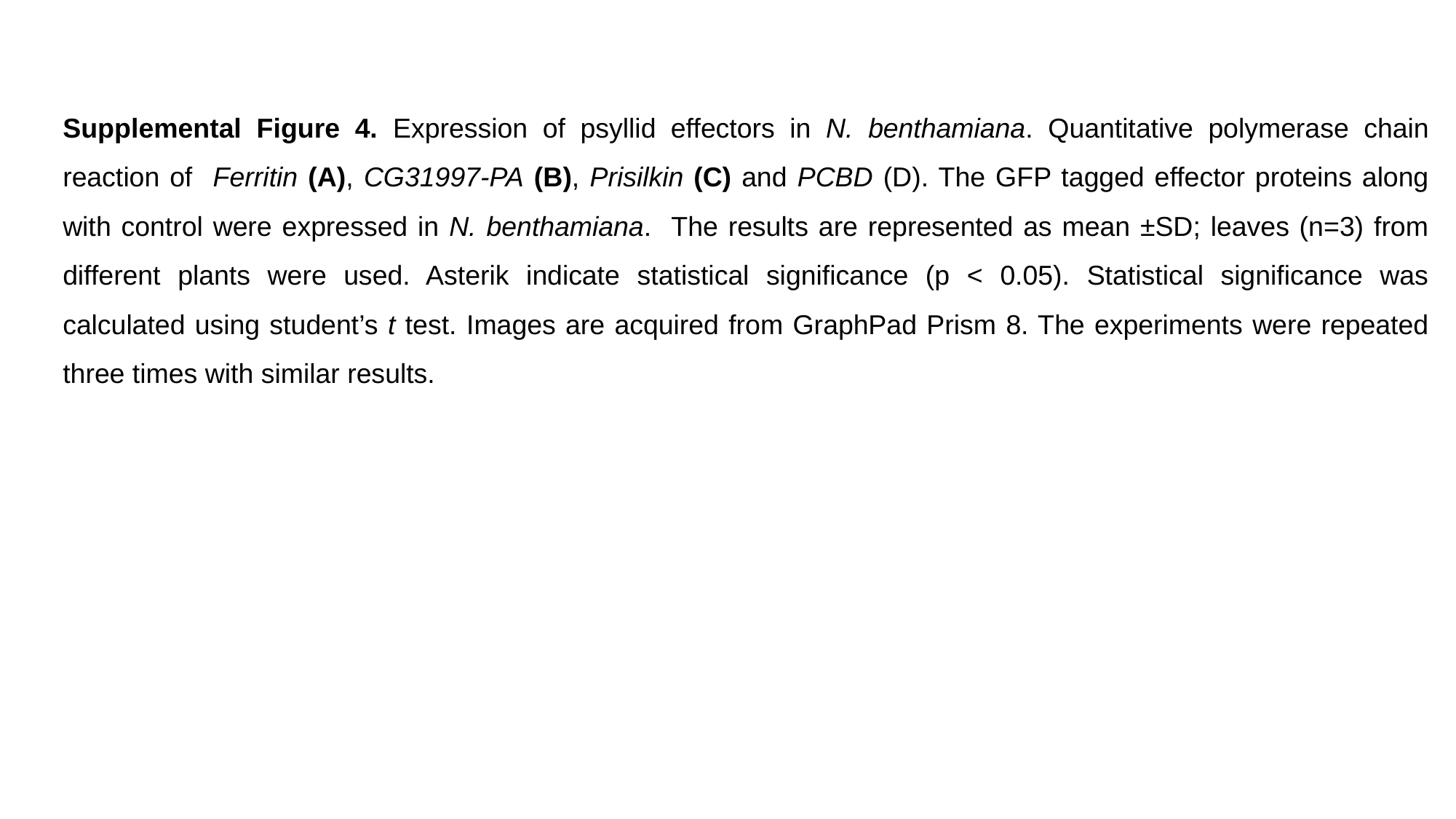

Supplemental Figure 4. Expression of psyllid effectors in N. benthamiana. Quantitative polymerase chain reaction of Ferritin (A), CG31997-PA (B), Prisilkin (C) and PCBD (D). The GFP tagged effector proteins along with control were expressed in N. benthamiana. The results are represented as mean ±SD; leaves (n=3) from different plants were used. Asterik indicate statistical significance (p < 0.05). Statistical significance was calculated using student’s t test. Images are acquired from GraphPad Prism 8. The experiments were repeated three times with similar results.

## Slide 8
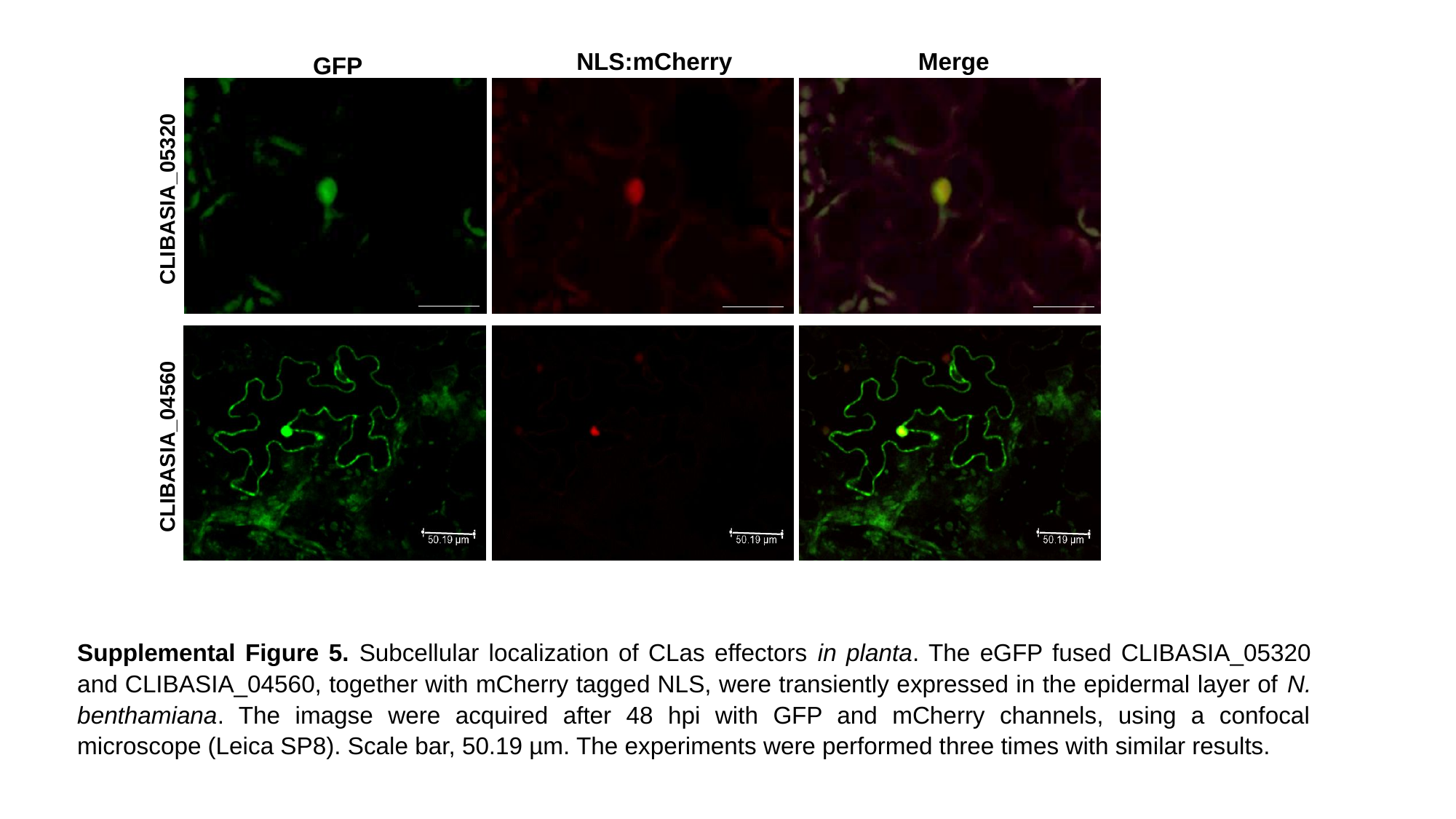

NLS:mCherry
Merge
GFP
CLIBASIA_05320
CLIBASIA_04560
Supplemental Figure 5. Subcellular localization of CLas effectors in planta. The eGFP fused CLIBASIA_05320 and CLIBASIA_04560, together with mCherry tagged NLS, were transiently expressed in the epidermal layer of N. benthamiana. The imagse were acquired after 48 hpi with GFP and mCherry channels, using a confocal microscope (Leica SP8). Scale bar, 50.19 µm. The experiments were performed three times with similar results.
